# Supplementary material for: Lateral hypothalamic glutamatergic inputs to VTA glutamatergic neurons mediate prioritization of innate defensive behavior over feeding
Source: Nat Commun. 2024 Jan 9;15:403. doi: 10.1038/s41467-023-44633-w (PMC10776608; doi:10.1038/s41467-023-44633-w)
Supplement: Supplementary file 2 — Supplementary information [file 41467_2023_44633_MOESM2_ESM.pdf]

Nature Communications

Supplementary information

Lateral hypothalamic glutamatergic inputs to VTA glutamatergic neurons mediate prioritization of innate defensive behavior over feeding

M. Flavia Barbano, PhD; Shiliang Zhang, PhD; Emma Chen, BS; Orlando Espinoza, BS; Uzma Mohammad, BA; Yocasta Alvarez-Bagnarol, PhD; Bing Liu, MD; Suyun Hahn, PhD; Marisela Morales, PhD

Supplementary Table 1. Average amount of food eaten by sated or food restricted mice, in grams, throughout the study

Figure	Group	2.5 Hz	5 Hz	10 Hz	20 Hz	
2B	eYFP	0.02 ± 0.01	0.03 ± 0.02	0.04 ± 0.02	0.03 ± 0.02	
	ChR2-eYFP	0.002 ± 0.002	0.00 ± 0.00	0.01 ± 0.01	0.00 ± 0.00	
**Figure**	**Group**	**No laser (0-3 min)**	**No laser (4-7 min)**	**Laser (0-3 min)**	**Laser (4-7 min)**	
2E	eYFP	0.05 ± 0.02	0.08 ± 0.04	0.06 ± 0.04	0.07 ± 0.03	
	ChR2-eYFP	0.17 ± 0.03	0.14 ± 0.01	0.00 ± 0.00	0.24 ± 0.02	
	Halo-eYFP	0.14 ± 0.02	0.15 ± 0.03	0.15 ± 0.03	0.15 ± 0.02	
	ChR2-eYFP (P)	0.17 ± 0.03	0.11 ± 0.02	0.00 ± 0.00	0.19 ± 0.03	
**Figure**	**Group**	**Baseline**	**No laser**	**Laser**		
3H	eYFP	0.30 ± 0.03	0.16 ± 0.03	0.15 ± 0.02		
	Halo-eYFP	0.34 ± 0.06	0.18 ± 0.02	0.28 ± 0.01		
**Figure**	**Group**	**Baseline**	**Rat**			
4N	mCherry	0.16 ± 0.02	0.09 ± 0.02			
	Caspase	0.31 ± 0.04	0.30 ± 0.05			
**Figure**	**Group**	**Baseline**	**Laser**	**Rat**	**Laser + rat**	
5J	mCherry	0.27 ± 0.03	0.008 ± 0.002	0.17 ± 0.02	0.012 ± 0.004	
	Caspase	0.30 ± 0.07	0.12 ± 0.06	0.39 ± 0.08	0.19 ± 0.06	
**Figure**	**Group**	**Day 1**	**Day 2**	**Day 3**	**Day 4**	**Day 5**
Sup. Fig. 10F	Male	0.20 ± 0.05	0.18 ± 0.05	0.30 ± 0.02	0.26 ± 0.03	0.21 ± 0.05
	Female	0.13 ± 0.03	0.14 ± 0.03	0.26 ± 0.02	0.24 ± 0.04	0.26 ± 0.04
**Figure**	**Group**	**Intake**				
Sup. Fig. 10H	eYFP	0.19 ± 0.04				
	ChR2-eYFP	0.08 ± 0.03				
**Figure**	**Group**	**No laser**	**No laser**			
Sup. Fig. 10J	eYFP	0.27 ± 0.03	0.22 ± 0.03			
	ChR2-eYFP	0.23 ± 0.05	0.03 ± 0.01			
	Halo-eYFP	0.26 ± 0.06	0.21 ± 0.04			
**Figure**	**Group**	**Baseline**	**No laser**	**Laser**		
Sup. Fig. 12J	eYFP	0.25 ± 0.03	0.07 ± 0.02	0.04 ± 0.01		
	Halo-eYFP	0.25 ± 0.09	0.11 ± 0.03	0.31 ± 0.06		
**Figure**	**Group**	**Day 1**	**Day 2**	**Day 3**		
Sup. Fig. 13C	mCherry	0.12 ± 0.02	0.12 ± 0.02	0.20 ± 0.03		
	Caspase	0.15 ± 0.02	0.15 ± 0.02	0.23 ± 0.03		
**Figure**	**Group**	**Day 1**	**Day 2**	**Day 3**		
Sup. Fig. 13G	mCherry	0.10 ± 0.01	0.13 ± 0.02	0.09 ± 0.01		
	Caspase	0.12 ± 0.03	0.17 ± 0.05	0.12 ± 0.05		
**Figure**	**Group**	**Day 1**	**Day 2**	**Day 3**		
Sup. Fig. 13K	mCherry	0.13 ± 0.02	0.11 ± 0.02	0.20 ± 0.02		
	Caspase	0.12 ± 0.02	0.12 ± 0.03	0.20 ± 0.04		
**Figure**	**Group**	**Baseline**	**Rat**			
Sup. Fig. 14I	mCherry	0.09 ± 0.01	0.03 ± 0.01			
	Caspase	0.22 ± 0.07	0.14 ± 0.06			
**Figure**	**Group**	**Baseline**	**Rat**			
Sup. Fig. 14R	mCherry	0.28 ± 0.04	0.17 ± 0.04			
	Caspase	0.29 ± 0.03	0.19 ± 0.04			

Data are presented as mean values ± SEM. Source data are provided as a Source Data file.

Supplementary Table 2. Statistical values for all the experiments reported in the study		
**Figure**	**Main effects**	**Interactions**
1D	Group: F(1,18)=1.74, p=0.20<br><br>Experimental phase: F(6,108)=1.62, p=0.15<br>*Chamber: F(2,36)=30.47, p<0.00001*	Group x experimental phase: F(6,108)=1.41, p=0.22<br>*Group x chamber: F(2,36)=94.71, p<0.00001*<br>*Experimental phase x chamber: F(12,216)=23.67, p<0.00001*<br>*Group x chamber x experimental phase: F(12,216)=21.46, p<0.00001*
1G	Chamber: F(2,12)=3.13, p=0.08<br><br>Experimental phase: F(2,12)=1.00, p=0.40	*Chamber x experimental phase: F(4,24)=5.09, p=0.004*
1H	*Chamber: F(2,14)=3.75, p=0.049*<br><br>Experimental phase: F(2,14)=-25.89, p=1	*Chamber x experimental phase: F(4,28)=43.47, p<0.00001*
1I	eYFP: X^2_9=0.00, p=1<br>*ChR2-eYFP: X^2_9=18.00, p=0.001*	
1J	*Treatment: F(1,4)=160.57, p=0.0002*<br><br>*Experimental phase: F(2,8)=35.44, p=0.0001*	*Treatment x experimental phase: F(2,8)=160.57, p<0.00001*
1K	Treatment: F(1,7)=0.85, p=0.39<br><br>Experimental phase: F(2,14)=3.18, p=0.07	Treatment x experimental phase: F(2,14)=0.86, p=0.45
2B	*Group: F(1,17)=11.06, p=0.004*<br>Frequency: F(3,51)=0.73, p=0.54	Group x frequency: F(3,51)=0.14, p=0.94
2D	*Group: F(3,31)=26.25, p<0.00001*<br><br>*Experimental phase: F(1,31)=77.61, p<0.00001*	*Group x experimental phase: F(3,31)=19.53, p<0.00001*
2E	Group: F(3,31)=1.94, p=0.14<br><br>*Experimental phase: F(1,31)=36.13, p<0.00001*	*Group x experimental phase: F(3,31)=16.21, p<0.00001*
2G	*Group: F(1,10)=12.81, p=0.005*<br><br>*Experimental phase: F(2,20)=14.58, p=0.0001*	*Group x experimental phase: F(2,20)=26.44, p<0.00001*
3E	*t(18)=-2.38, p=0.03*	
3H	Group: F(1,10)=2.57, p=0.14<br><br>*Experimental phase: F(2,20)=20.95, p=0.00001*	*Group x experimental phase: F(2,20)=3.52, p=0.049*
4E	*t(14)=-2.40, p=0.03*	
4K	*t(4)=8.39, p=0.0011*	
4N	*t(22)=-3.08, p=0.005*	
5G	*Zone: F(1,15)=1520.10, p<0.00001*	Zone x group: F(1,15)=0.97, p=0.34<br>Zone x experimental phase: F(2,30)=0.63, p=0.54<br>Zone x group x experimental phase: F(2,30)=0.07, p=0.93
5H	Group: F(1,15)=0.01, p=0.92	*Group x experimental phase: F(2,30)=16.47, p=0.00001*

	Experimental phase: F(2,30)=26.09, p<0.00001	
5I	Group: F(1,15)=0.02, p=0.89<br>*Experimental phase: F(2,30)=18.64, p=0.00001*	*Group x experimental phase: F(2,30)=10.63, p=0.0003*
5J	*Group: F(1,19)=5.92, p=0.025*<br>*Experimental phase: F(3,57)=30.77, p<0.00001*	*Group x experimental phase: F(3,57)=3.66, p=0.02*
Sup. 2M	*Group: F(2,6)=26.92, p=0.001*	
Sup. 2N	*Group: F(2,6)=26.92, p=0.001*<br>*Bregma: F(8,48)=7.26, p<0.00001*	*Group x bregma: F(16,48)=3.26, p=0.0008*
Sup. 3B	Pulse width: F(3,24)=2.15, p=0.12<br>*Frequency: F(4,32)=3.94, p=0.01*	Pulse width x frequency: F(12,96)=1.08, p=0.39
Sup. 3C	Group: F(1,18)=0.72, p=0.41<br>*Day: F(9,162)=5.17, p<0.00001*	*Group x day: F(9,162)=2.36, p=0.02*
Sup. 3D	*Group: F(1,18)=4.85, p=0.04*<br>*Day: F(1,18)=6.77, p=0.002*<br>Wheel: F(1,18)=0.20, p=0.66	Group x day: F(1,18)=0.23, p=0.64<br>*Wheel x group: F(1,18)=6.47, p=0.002*<br>Wheel x day: F(1,18)=0.10, p=0.75<br>Wheel x group x day: F(1,18)=3.69, p=0.07
Sup. 4D	Group: F(1,16)=0.06, p=0.81<br>Experimental phase: F(6,96)=1.03, p=0.41<br>*Chamber: F(2,32)=23.75, p<0.00001*	Group x experimental phase: F(6,96)=0.27, p=0.93<br>*Group x chamber: F(2,32)=23.41, p<0.00001*<br>*Experimental phase x chamber: F(10,160)=4.88, p<0.00001*<br>*Group x chamber x experimental phase: F(10,160)=6.71, p<0.00001*
Sup. 4E	Group: F(1,15)=0.42, p=0.53<br>Experimental phase: F(5,75)=0.55, p=0.74<br>*Chamber: F(2,30)=25.63, p<0.00001*	Group x experimental phase: F(5,75)=0.55, p=0.74<br>Group x chamber: F(2,30)=0.89, p=0.42<br>*Experimental phase x chamber: F(10,150)=2.49, p=0.009*<br>Group x chamber x experimental phase: F(10,150)=1.61, p=0.11
Sup. 5B	Experimental phase: F(3,12)=-49.68, p=1<br>*Chamber: F(2,8)=10.59, p=0.006*	*Chamber x experimental phase: F(6,24)=16.79, p<0.00001*
Sup. 5C	*Experimental phase: F(3,15)=10.05, p=0.0007*<br>*Chamber: F(2,10)=4.94, p=0.03*	*Chamber x experimental phase: F(6,30)=19.68, p<0.00001*
Sup. 6C	*Group: F(1,16)=5.85, p=0.03*<br>*Zone: F(1,16)=9.83, p=0.006*	*Group x zone: F(1,16)=13.98, p=0.002*
Sup. 6D	*Group: F(1,16)=8.45, p=0.01*<br>*Zone: F(1,16)=13.51, p=0.002*	*Group x zone: F(1,16)=10.91, p=0.005*
Sup. 6E	Group: F(1,16)=2.49, p=0.13<br>*Zone: F(1,16)=9.53, p=0.007*	*Group x zone: F(1,16)=8.32, p=0.01*
Sup. 6F	Group: F(1,16)=0.55, p=0.47<br>*Zone: F(1,16)=14.51, p=0.002*	*Group x zone: F(1,16)=11.71, p=0.004*
Sup. 6G	Group: F(1,16)=0.87, p=0.36<br>Zone: F(1,16)=0.87, p=0.36	Group x zone: F(1,16)=2.38, p=0.14

Sup. 6H	Group: F(1,16)=1.33, p=0.26<br>Zone: F(1,16)=0.93, p=0.35	Group x zone: F(1,16)=0.76, p=0.40
Sup. 7B	*Zone: F(1,29)=1478.90, p<0.00001*	Zone x group: F(2,29)=1.48, p=0.25<br>*Zone x experimental phase: F(2,58)=3.00, p=0.06*<br>Zone x group x experimental phase: F(2,30)=0.87, p=0.49
Sup. 7C	*Group: F(2,29)=18.58, p=0.00001*<br>*Experimental phase: F(2,58)=23.99, p<0.00001*	*Group x experimental phase: F(4,58)=23.19, p<0.00001*
Sup. 7D	*Group: F(2,29)=17.84, p=0.00001*<br>*Experimental phase: F(2,58)=23.41, p<0.00001*	*Group x experimental phase: F(4,58)=22.79, p<0.00001*
Sup. 7E	Group: F(2,29)=1.52, p=0.24<br>*Experimental phase: F(2,58)=5.99, p=0.004*	*Group x experimental phase: F(4,58)=3.33, p=0.02*
Sup. 7F	Group: F(2,29)=1.75, p=0.19<br>*Experimental phase: F(2,58)=5.63, p=0.006*	*Group x experimental phase: F(4,58)=4.74, p=0.002*
Sup. 7G	Group: F(1,18)=1.27, p=0.28<br>Experimental phase: F(2,36)=2.26, p=0.12	Group x experimental phase: F(2,36)=1.64, p=0.21
Sup. 7H	Group: F(1,18)=1.27, p=0.28<br>Experimental phase: F(2,36)=2.26, p=0.12	Group x experimental phase: F(2,36)=1.64, p=0.21
Sup. 8C	t(16)=0.08, p=0.94	
Sup. 8D	t(16)=0.08, p=0.94	
Sup. 8E	Group: F(1,16)=1.57, p=0.23<br>*Zone: F(1,16)=7464.90, p<0.00001*	*Group x zone: F(1,16)=5.92, p=0.03*
Sup. 8F	Group: F(1,16)=0.48, p=0.50<br>*Zone: F(1,16)=19.88, p=0.0004*	Group x zone: F(1,16)=0.96, p=0.34
Sup. 9B	*Group: F(1,16)=15.17, p=0.001*<br>*Experimental phase: F(2,32)=61.04, p<0.00001*	*Group x experimental phase: F(2,32)=48.37, p<0.00001*
Sup. 9C	*Group: F(1,16)=14.97, p=0.001*<br>*Time point: F(5,80)=47.30, p<0.00001*	*Group x experimental phase: F(5,80)=37.13, p<0.00001*
Sup. 9D	*Group: F(1,16)=14.94, p=0.001*<br>*Experimental phase: F(2,32)=60.91, p<0.00001*	*Group x experimental phase: F(2,32)=48.23, p<0.00001*
Sup. 9E	*Group: F(1,16)=27.17, p=0.00009*<br>*Experimental phase: F(2,32)=21.73, p<0.00001*<br>*Zone: F(1,16)=29.44, p=0.00006*	*Group x experimental phase: F(2,32)=34.73, p<0.00001*<br>Zone x group: F(1,16)=0.34, p=0.57<br>Zone x experimental phase: F(2,32)=2.01, p=0.15<br>Zone x group x experimental phase: F(2,32)=0.25, p=0.78
Sup. 9G	*U=13.50, p=0.02*	

Sup. 9H	*U=0, p=0.0004*	
Sup. 10B	eYFP: X^2_9=7.00, p=0.64<br>*ChR2-eYFP: X^2_9=9.00, p=0.44*	
Sup. 10C	eYFP: X^2_9=6.37, p=0.70<br>*ChR2-eYFP: X^2_9=9.00, p=0.44*	
Sup. 10D	eYFP: X^2_9=9.30, p=0.41<br>*ChR2-eYFP: X^2_9=9.00, p=0.44*	
Sup. 10E	eYFP: X^2_9=7.57, p=0.58<br>*ChR2-eYFP: X^2_9=9.00, p=0.44*	
Sup. 10F	Sex: F(1,22)=0.53, p=0.48<br>*Day: F(4,88)=5.59, p=0.0005*	Sex x day: F(4,88)=1.12, p=0.35
Sup. 10G	*Group: F(1,10)=43.36, p=0.00006*<br>Trial: F(9,90)=1.55, p=0.14	*Group x trial: F(9,90)=2.02, p=0.045*
Sup. 10H	*t(10)=-2.36, p=0.04*	
Sup. 10I	*Group: F(2,29)=46.08, p<0.00001*	
Sup. 10J	*Group: F(2,29)=8.16, p=0.002*	
Sup. 11F	t(24)=1.15, p=0.26	
Sup. 11G	t(24)=0.57, p=0.57	
Sup. 11H	t(24)=-0.35, p=0.73	
Sup. 11I	t(24)=0.70, p=0.49	
Sup. 12E	*t(7)=-4.60, p=0.002*	
Sup. 12H	t(7)=-0.05, p=0.96	
Sup. 12I	*Stimulus: F(1,7)=7.27, p=0.031*<br>Zone: F(1,7)=5.51, p=0.05	*Stimulus x zone: F(1,7)=16.28, p=0.005*
Sup. 12J	Group: F(1,16)=3.47, p=0.08<br>*Experimental phase: F(2,32)=16.29, p=0.00001*	*Group x experimental phase: F(2,32)=12.91, p=0.00008*
Sup. 13B	*Group: F(1,4)=70.38, p=0.001*<br>*Bregma: F(6,24)=2.99, p=0.03*	*Group x bregma: F(6,24)=2.68, p=0.04*
Sup. 13C	Group: F(1,22)=1.47, p=0.24<br>*Day: F(2,44)=7.83, p=0.001*	Group x day: F(2,44)=0.009, p=0.99
Sup. 13D	t(22)=-0.27, p=0.79	
Sup. 13F	*Group: F(1,4)=26.03, p=0.007*<br>*Bregma: F(6,24)=10.30, p=0.00001*	*Group x bregma: F(6,24)=4.72, p=0.003*
Sup. 13G	Group: F(1,17)=0.48, p=0.50<br>*Day: F(2,34)=4.80, p=0.015*	Group x day: F(2,34)=0.15, p=0.86
Sup. 13H	t(17)=0.57, p=0.58	
Sup. 13J	*Group: F(1,4)=59.29, p=0.002*<br>*Bregma: F(6,24)=3.78, p=0.009*	Group x bregma: F(6,24)=0.79, p=0.59
Sup. 13K	Group: F(1,15)=0.01, p=0.91<br>*Day: F(2,30)=11.27, p=0.0002*	Group x day: F(2,30)=0.17, p=0.85
Sup. 13L	t(15)=0.57, p=0.58	
Sup. 14F	*t(4)=5.10, p=0.007*	
Sup. 14I	t(16)=-0.72, p=0.48	
Sup. 14O	*t(4)=7.70, p=0.002*	
Sup. 14R	t(15)=-0.93, p=0.36	

Significant main effects or interactions are depicted in italic.

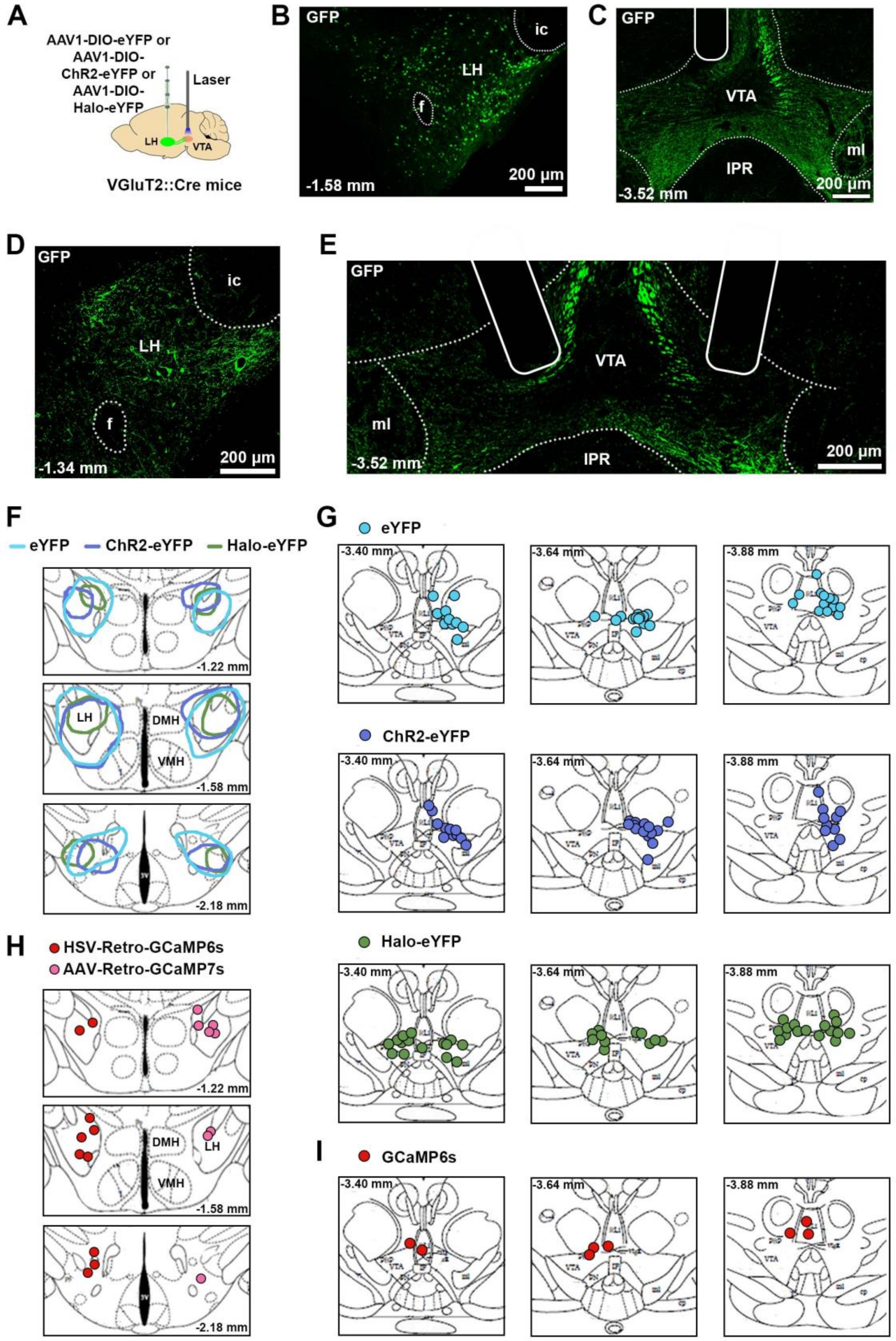

Supplementary Figure 1. Detection of viral injection sites and location of optical probes. A. LH injection of AAV1-DIO-eYFP, AAV1-DIO-ChR2-eYFP, or AAV1-DIO-Halo-eYFP and VTA optic fibers. **B.** Expression of ChR2-eYFP (green) in LH neurons. **C.** VTA optic fiber placement (solid line) and detection of eYFP fibers from LH-VGluT2 neurons (green). **D.** Expression of Halo-eYFP (green) in LH neurons. **E.** VTA bilateral optic fiber placement (solid line) and detection of eYFP fibers from LH-VGluT2 neurons (green). **F.** Rostro-caudal extension of viral injections (eYFP, ChR2-eYFP and Halo-eYFP) within the LH. **G.** VTA optic fiber placements in eYFP (top), ChR2-eYFP (middle), and Halo-eYFP (bottom) mice. **H.** Photometry fiber placements within the LH. **I.** Photometry fiber placements within the VTA. Cp: cerebral peduncle; DMH: dorsomedial hypothalamus; f: fornix; GFP: green fluorescent protein; ic: internal capsule; IF: interfascicular nucleus; IPR: interpeduncular nucleus, rostral subnucleus; LH: lateral hypothalamus; ml: medial lemniscus; mtg: mammillotegmental tract; PBP: parabrachial pigmented nucleus; PN: paranigral nucleus; Rli: rostro linear nucleus; VTA: ventral tegmental area; vtgx: ventral tegmental decussation.

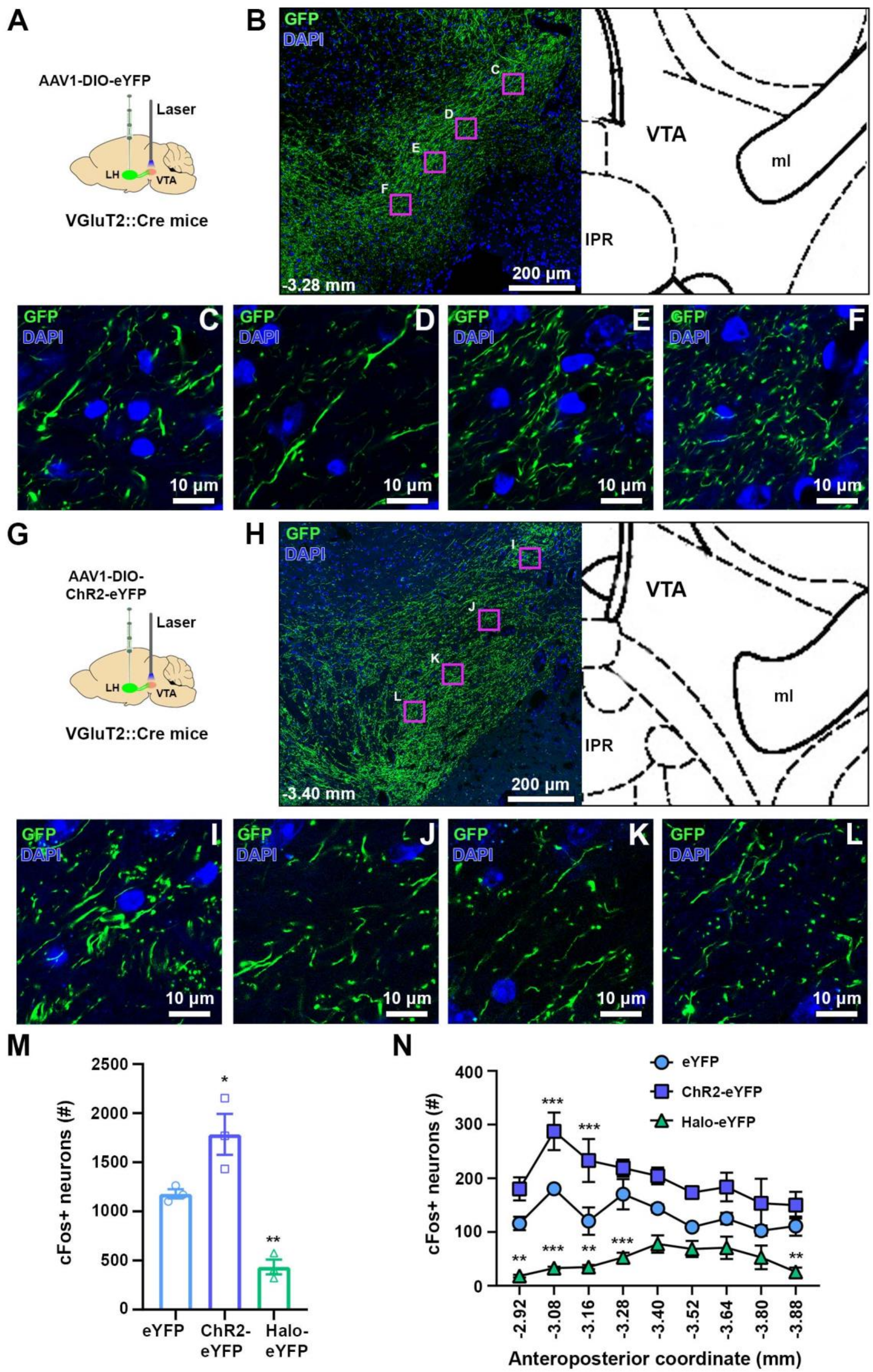

Supplementary Figure 2. Expression of eYFP in VTA neurons and cFos induction after photostimulation or photoinhibition. A. LH injection of AAV1-DIO-eYFP and VTA optic fiber. **B.** VTA detection of eYFP fibers from LH-VGluT2 neurons (green). **C-F.** High magnification of squares in B showing lack of somatic eYFP labeling within the VTA. **G.** LH injection of AAV1-DIO-ChR2-eYFP and VTA optic fiber. **H.** VTA detection of ChR2-eYFP fibers from LH-VGluT2 neurons (green). **I-L.** High magnification of squares in **H** showing lack of somatic eYFP labeling within the VTA. IPR: interpeduncular nucleus; ml: medial lemniscus; VTA: ventral tegmental area. **M.** VTA total number of cFos+ neurons was higher in ChR2-eYFP mice and lower in Halo-eYFP mice after laser administration (n=3 per experimental group, 9 sections/mouse; group: $F_{(2,6)}$=26.92, P<0.01, ANOVA with Newman-Keuls post hoc test). * P<0.05, ** P<0.01, against eYFP mice. **N.** Anteroposterior distribution of cFos+ neurons within the VTA after laser administration (group x bregma: $F_{(16,48)}$=3.26, P<0.001, ANOVA with Newman-Keuls post hoc test). * P<0.05, ** P<0.01, *** P<0.001, against eYFP mice. Data are presented as mean values ± SEM. Source data are provided as a Source Data file.

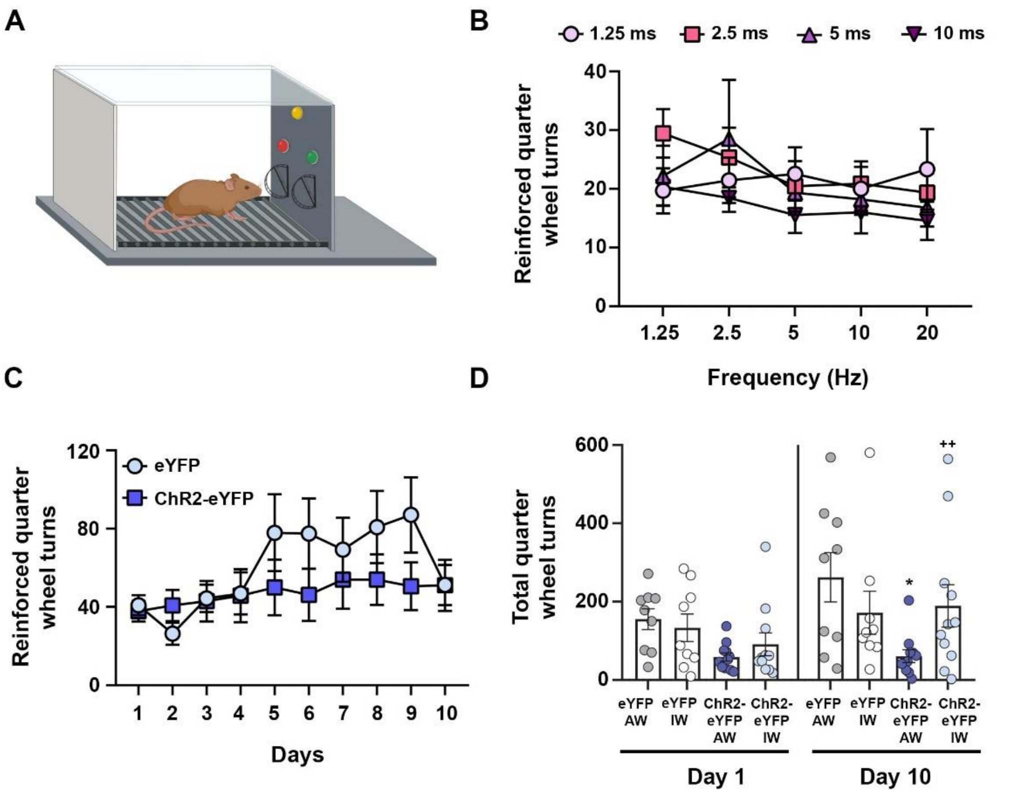

Supplementary Figure 3. VTA photostimulation of LH-VGluT2 fibers is not reinforcing. A. Operant chamber featured with two wheels for VTA optical intracranial self-stimulation (oICSS) of LH-VGluT2 fibers. Quarter turns of the active wheel resulted in 2 s train of photostimulation at varying frequencies, while quarter turns on the inactive wheel did not produce any scheduled consequence. Created with BioRender.com. **B.** oICSS was not observed in ChR2-eYFP mice (n=11) tested at variable pulse durations and frequencies (pulse width x frequency: $F_{(12,96)}$=1.08, P=0.39, n.s., ANOVA). Reinforced quarter wheel turns, which resulted in photostimulation trains, were recorded in 10-min sessions. **C.** Similar number of quarter wheel turns, which resulted in VTA photostimulations at 20 Hz in a 30-min session, were obtained by ChR2-eYFP (n=11) and eYFP control (n=9) mice (group: $F_{(1,18)}$=0.72, P=0.41, n.s., ANOVA). **D.** The number of total quarter wheel turns (reinforced and non-reinforced) on the active (AW) and inactive (IW) wheels in 30-min sessions across days was significantly reduced in ChR2-eYFP (n=11) mice when compared to eYFP control mice (n=9; group x wheel: $F_{(1,18)}$=6.47, P<0.05, ANOVA with Newman-Keuls post hoc test) * P<0.05, against eYFP for the same wheel, ++ P<0.01, against active wheel for the same group. Data are presented as mean values ± SEM. Source data are provided as a Source Data file.

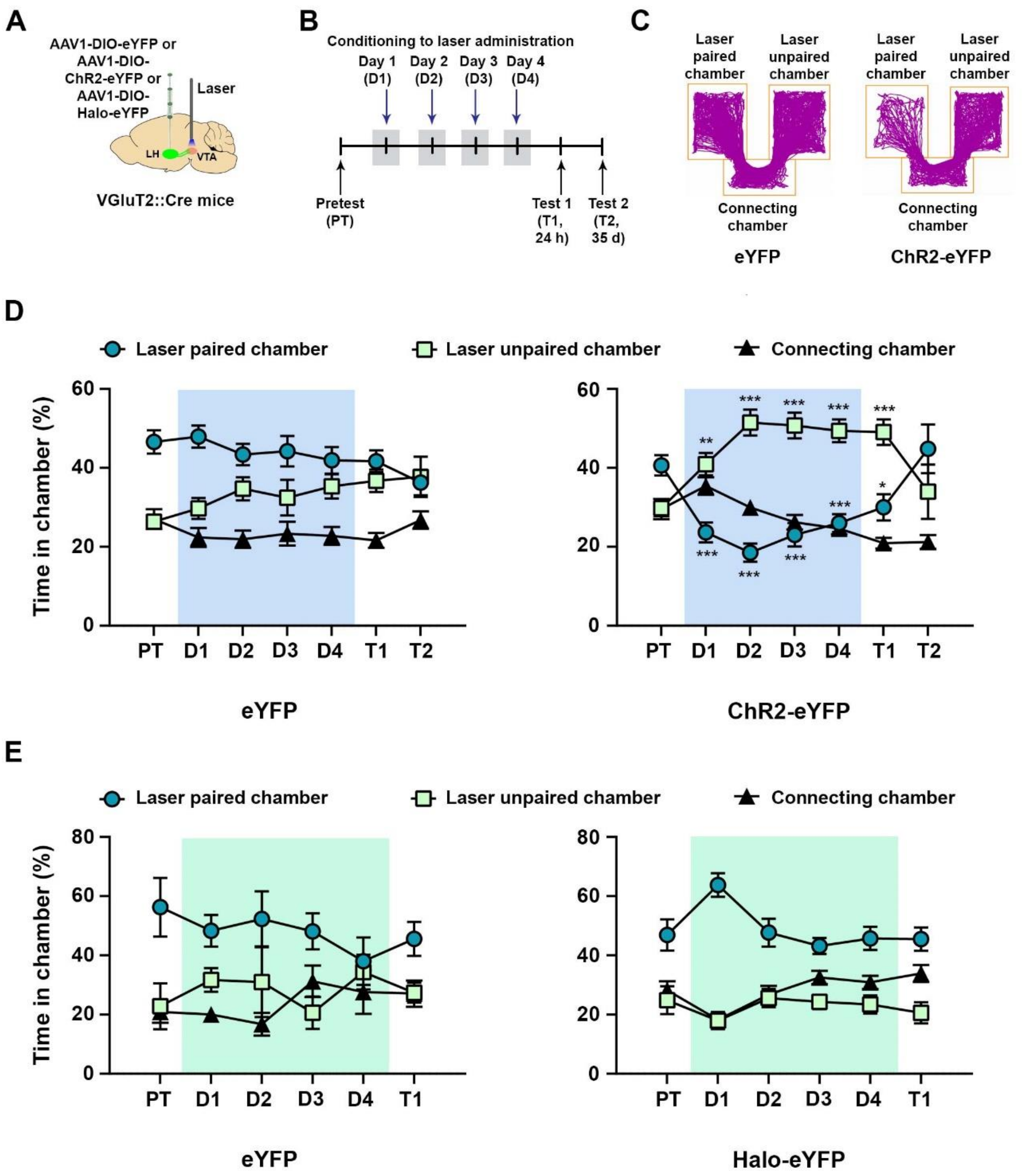

Supplementary Figure 4. VTA photostimulation of LH-VGluT2 fibers is aversive. A. LH injection of AAV1-DIO-eYFP, AAV1-DIO-ChR2-eYFP or AAV1-DIO-Halo-eYFP and VTA optic fiber. **B.** Timeline for place conditioning experiments. **C.** Track plot for an eYFP (left) and a ChR2-eYFP (right) mouse during the first day of photostimulation conditioning at 2.5 Hz (D1). **D.** ChR2-eYFP mice (n=18) spent significantly less time in the laser paired chamber at a stimulation frequency of 2.5 Hz than eYFP control mice (n=15) during and 24 h after photostimulation sessions, but not 35 days post-conditioning sessions (group x chamber x experimental phase: $F_{(12,156)}=1.90$, $P<0.05$, ANOVA with Newman-Keuls post hoc test). Light-blue rectangles indicate photostimulation. **E.** Lack of preference or avoidance to the laser paired chamber by both Halo-eYFP mice (n=11) and eYFP control mice (n=6) during VTA photoinhibition of LH-VGluT2 fibers ($F_{(10,150)}=1.61$, $P=0.11$, n.s., ANOVA). Green rectangles indicate photoinhibition. * P<0.05, ** P<0.01, *** P<0.001, against pretest. Data are presented as mean values ± SEM. Source data are provided as a Source Data file.

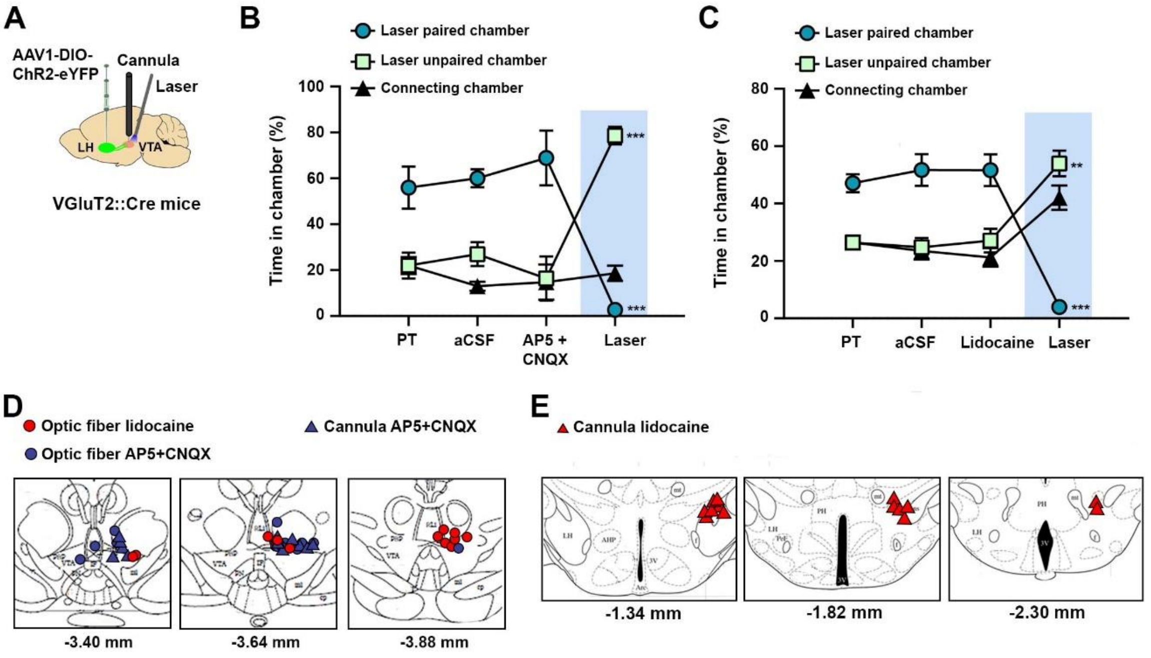

Supplementary Figure 5. Intra-VTA glutamatergic receptor antagonists or lidocaine do not induce aversion or escape attempts. A. LH injection of AAV1-DIO-ChR2-eYFP, intra-VTA microinjections and VTA optic fiber. **B.** NMDA (AP5) and AMPA (CNQX) receptor antagonists do not induce active avoidance responses on their own in ChR2-eYFP mice (n=5, chamber x experimental phase: $F_{(6,24)}$=16.79, P<0.001, ANOVA with Newman-Keuls post hoc test). *** P<0.001, against pretest or aCSF. **C.** Lidocaine does not induce active avoidance responses on its own in ChR2-eYFP mice (n=6, chamber x experimental phase: $F_{(6,30)}$=19.68, P<0.001, ANOVA with Newman-Keuls post hoc test). ** P<0.01, *** P<0.001, against pretest or aCSF. **D.** VTA placement of optic fibers and cannulae for pharmacological studies. cp: cerebral peduncle; IP: interpeduncular nucleus; ml: medial lemniscus; PBP: parabrachial pigmented nucleus; PN: paranigral nucleus; RLi: rostrolinear nucleus of the raphe; VTA: ventral tegmental area. **E.** LH placement of cannulae for lidocaine studies. AHP: anterior hypothalamic area, posterior part; Arc: arcuate nucleus; f: fornix; LH: lateral hypothalamus; mt: mammillothalamic tract; ns: nigrostriatal bundle; PeF: perifornical area; PH: posterior hypothalamic area; 3V: third ventricle. Light-blue rectangles indicate photostimulation. Data are presented as mean values ± SEM. Source data are provided as a Source Data file.

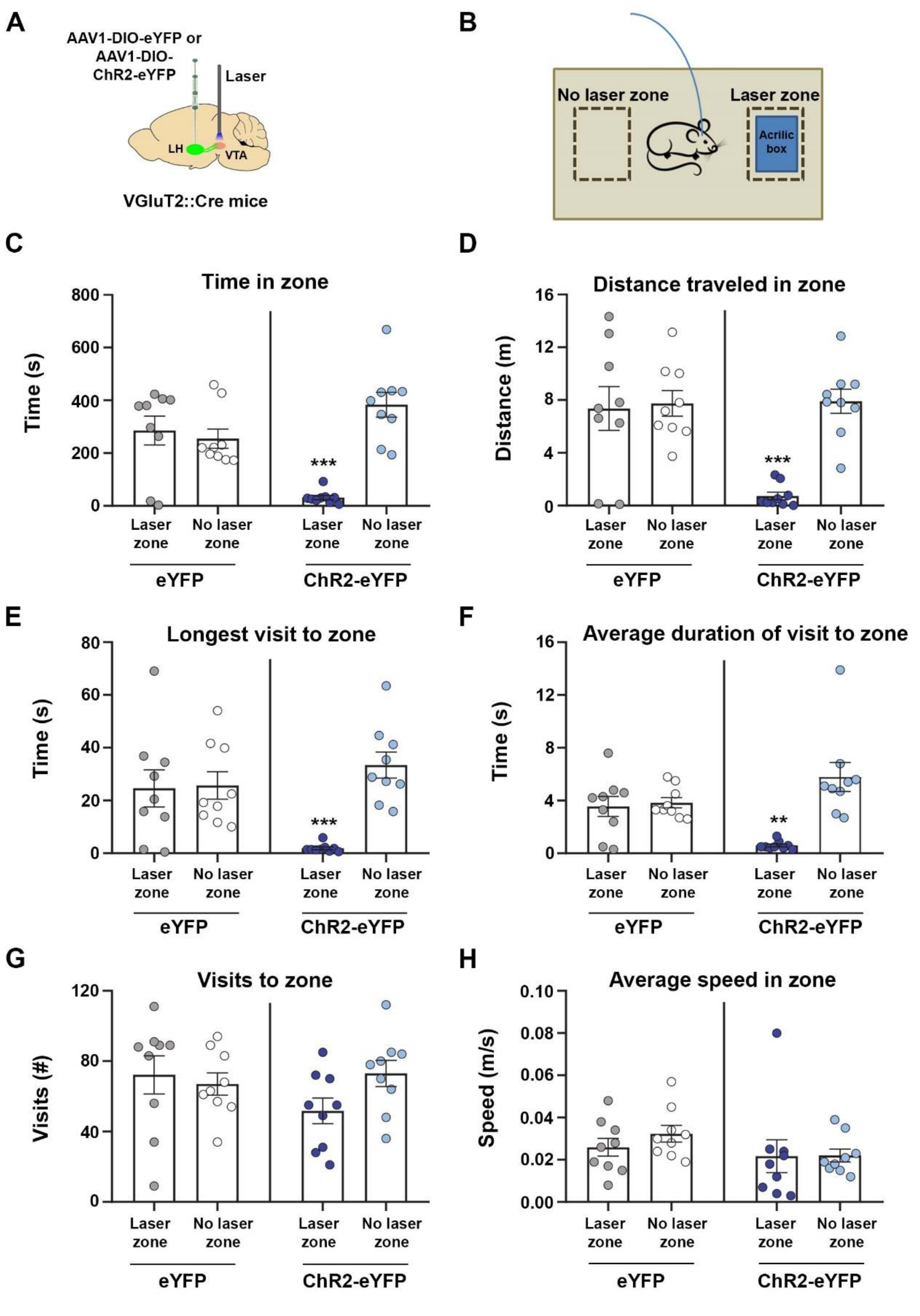

Supplementary Figure 6. VTA photostimulation of LH-VGluT2 fibers does not induce defensive burying. A. LH injection of AAV1-DIO-eYFP or AAV1-DIO-ChR2-eYFP and VTA optic fiber. **B.** Modified defensive burying paradigm in which mice experienced VTA photostimulation of LH-VGluT2 fibers each time they entered the acrylic box area (laser zone). **C.** ChR2-eYFP mice (n=9) spent significantly less time in the laser zone than eYFP control mice (n=9; group x zone: $F_{(1,16)}$=13.98, P<0.01, ANOVA with Newman-Keuls post hoc test). **D.** ChR2-eYFP mice (n=9) traveled significantly less distance in the laser zone than eYFP control mice (n=9; group x zone: $F_{(1,16)}$=10.91, P<0.01, ANOVA with Newman-Keuls post hoc test). **E.** The longest visit to the laser zone was significantly shorter for ChR2-eYFP mice (n=9) than for eYFP control mice (n=9; group x zone: $F_{(1,16)}$=8.32, P<0.05, ANOVA with Newman-Keuls post hoc test). **F.** The average duration of each visit to the laser zone was significantly shorter for ChR2-eYFP mice (n=9) than for eYFP control mice (n=9; group x zone: $F_{(1,16)}$=11.71, P<0.01, ANOVA with Newman-Keuls post hoc test). **G.** The total number of visits to each zone was not different between ChR2-eYFP (n=9) and eYFP control mice (n=9; group x zone: $F_{(1,16)}$=2.38, P=0.14, n.s., ANOVA). **H.** The average speed in each zone was not different between ChR2-eYFP (n=9) and eYFP control mice (n=9; group x zone: $F_{(1,16)}$=0.76, P=0.40, n.s., ANOVA). Asterisks indicate significant differences between groups for a given zone. ** P<0.01, *** P<0.001. Data are presented as mean values ± SEM. Source data are provided as a Source Data file.

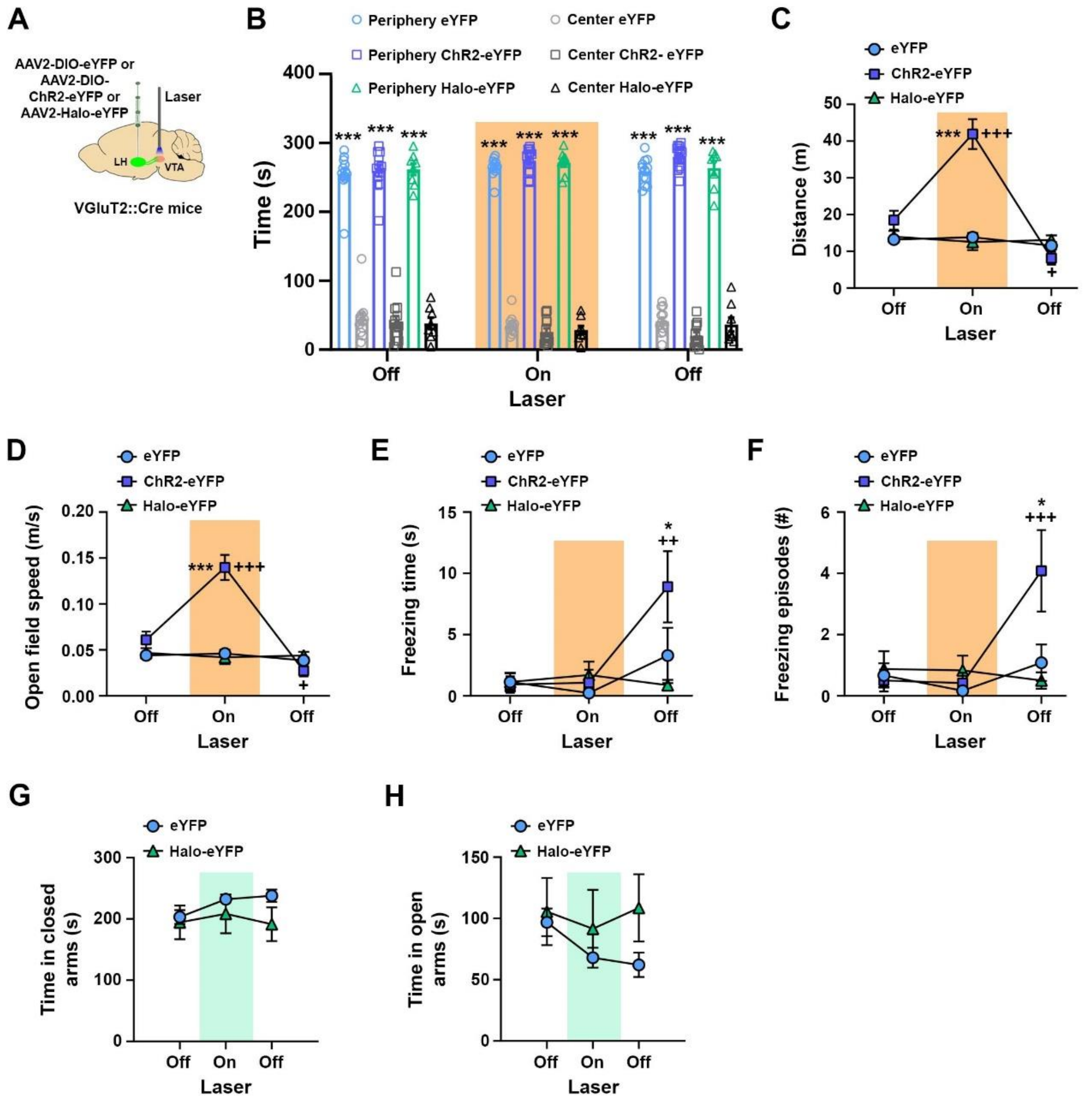

Supplementary Figure 7. VTA photostimulation or photoinhibition of LH-VGluT2 fibers do not play a role in anxiety-like or freezing behaviors. A. LH injection of AAV2-DIO-eYFP, AAV2-DIO-ChR2-eYFP or AAV2-DIO-Halo-eYFP and VTA optic fiber. **B.** Time spent in the periphery and center zones of an open field arena were similar between eYFP (n=12), ChR2-eYFP (n=12) and Halo-eYFP (n=12) mice before, during and after VTA photostimulation of LH-VGluT2 fibers (group x zone x experimental phase: $F_{(4,58)}$=0.87, P=0.49, n.s., ANOVA). *** P<0.001, against center for each experimental group. **C.** ChR2-eYFP mice (n=12) moved more than eYFP control (n=12) or Halo-eYFP (n=8) mice during VTA laser administration (group x experimental phase: $F_{(4,58)}$=23.19, P<0.001, ANOVA with Newman-Keuls post hoc test). *** P<0.001, against eYFP mice; + P<0.05, +++ P<0.001, against the first period of laser off for each experimental group. **D.** ChR2-eYFP mice (n=12) moved significantly faster than eYFP control (n=12) or Halo-eYFP (n=8) mice during VTA laser administration (group x experimental phase: $F_{(4,58)}$=22.79, P<0.001, ANOVA with Newman-Keuls post hoc test). *** P<0.001, against eYFP mice; + P<0.05, +++ P<0.001, against the first period of laser off for each experimental group. **E.** Time freezing during VTA laser administration was similar between eYFP control (n=12), ChR2-eYFP (n=12) and Halo-eYFP (n=8) mice (group: $F_{(2,29)}$=1.52, P=0.24; group x experimental phase: $F_{(4,58)}$=3.33, P<0.05, ANOVA with Newman-Keuls post hoc test). * P<0.05, against eYFP mice; ++ P<0.01, against the first period of laser off for each experimental group. **F.** Number of freezing episodes during VTA laser administration was similar between eYFP control (n=12), ChR2-eYFP (n=12) and Halo-eYFP (n=8) mice (group: $F_{(2,29)}$=1.75, P=0.19; group x experimental phase: $F_{(4,58)}$=4.74, P<0.01, ANOVA with Newman-Keuls post hoc test). * P<0.05, against eYFP mice; +++ P<0.001, against the first period of laser off for each experimental group. **G-H.** Anxiety-like behavior, expressed as the time spent in the closed (**G**) or open (**H**) arms of an elevated plus maze apparatus was similar between eYFP control (n=12) and Halo-eYFP (n=8) mice (group x experimental phase: $F_{(2,36)}$=1.64, P=0.21, n.s., ANOVA). Orange rectangles indicate either photostimulation or photoinhibition. Green rectangles indicate photoinhibition. Data are presented as mean values ± SEM. Source data are provided as a Source Data file.

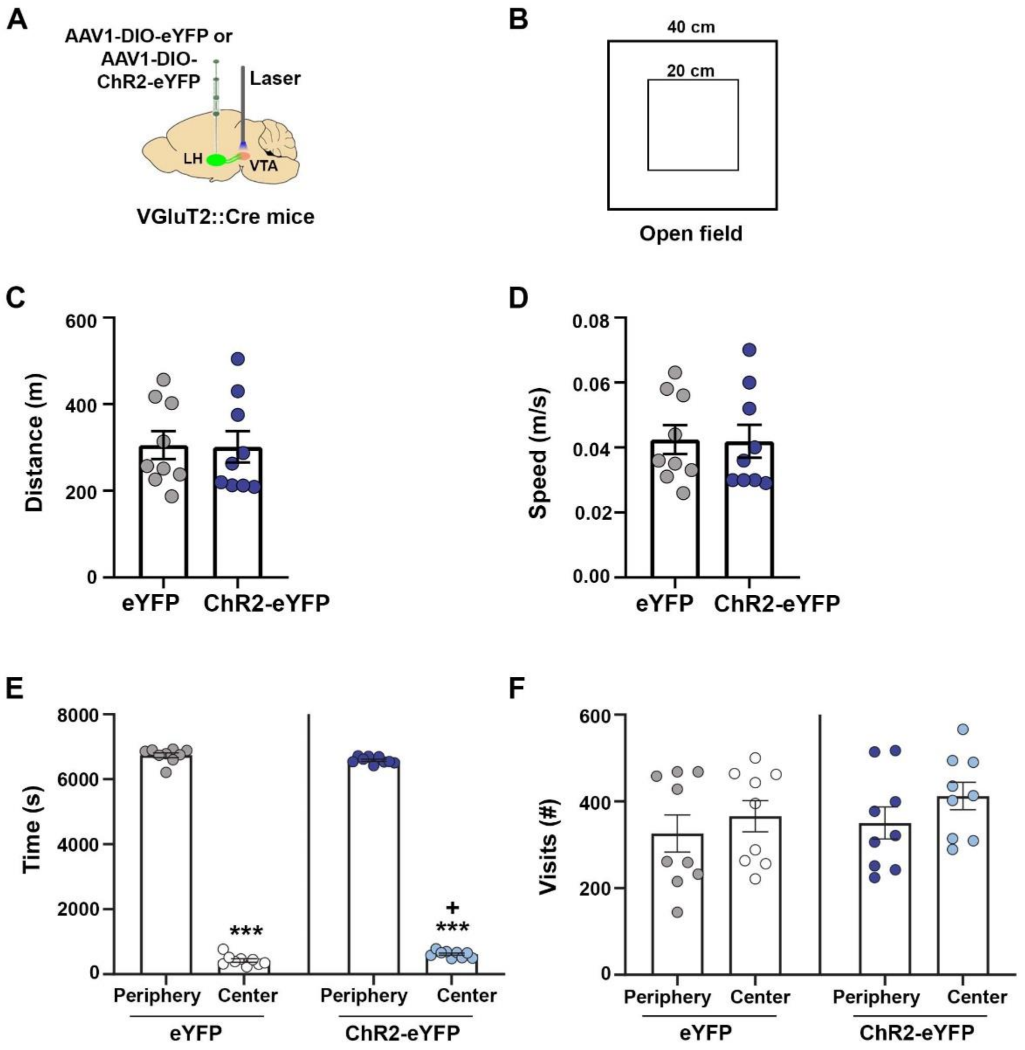

Supplementary Figure 8. Baseline locomotion and anxiety levels were similar between ChR2-eYFP and eYFP control mice during open field test habituation. A. LH injection of AAV1-DIO-eYFP or AAV1-DIO-ChR2-eYFP and VTA optic fiber. **B.** Anxiety and locomotion were measured in an open field arena. **C.** ChR2-eYFP (n=9) and eYFP control mice (n=9) travelled similar distances during a 2-h open field habituation test without photostimulation ($t_{(16)}$=0.08, P=0.94, n.s., two-tailed t test). **D.** ChR2-eYFP (n=9) and eYFP control mice (n=9) displayed similar speed during a 2-h open field habituation test without photostimulation ($t_{(16)}$=0.08, P=0.94, n.s., two-tailed t test). **E.** ChR2-eYFP (n=9) and eYFP control mice (n=9) spent equivalent amounts of time in the periphery and center zones of the open field arena during a 2-h habituation test without photostimulation (group: $F_{(1,16)}$=1.57, P=0.23, n.s., ANOVA with Newman-Keuls post hoc test). Asterisks indicate significant differences between center and periphery zones, plus sign indicates differences between groups. *** P<0.001; + P<0.05. **F.** The total number of visits to each zone was not different between ChR2-eYFP (n=9) and eYFP control mice (n=9; group x zone: $F_{(1,16)}$=1.57, P=0.23, n.s., ANOVA). Data are presented as mean values ± SEM. Source data are provided as a Source Data file.

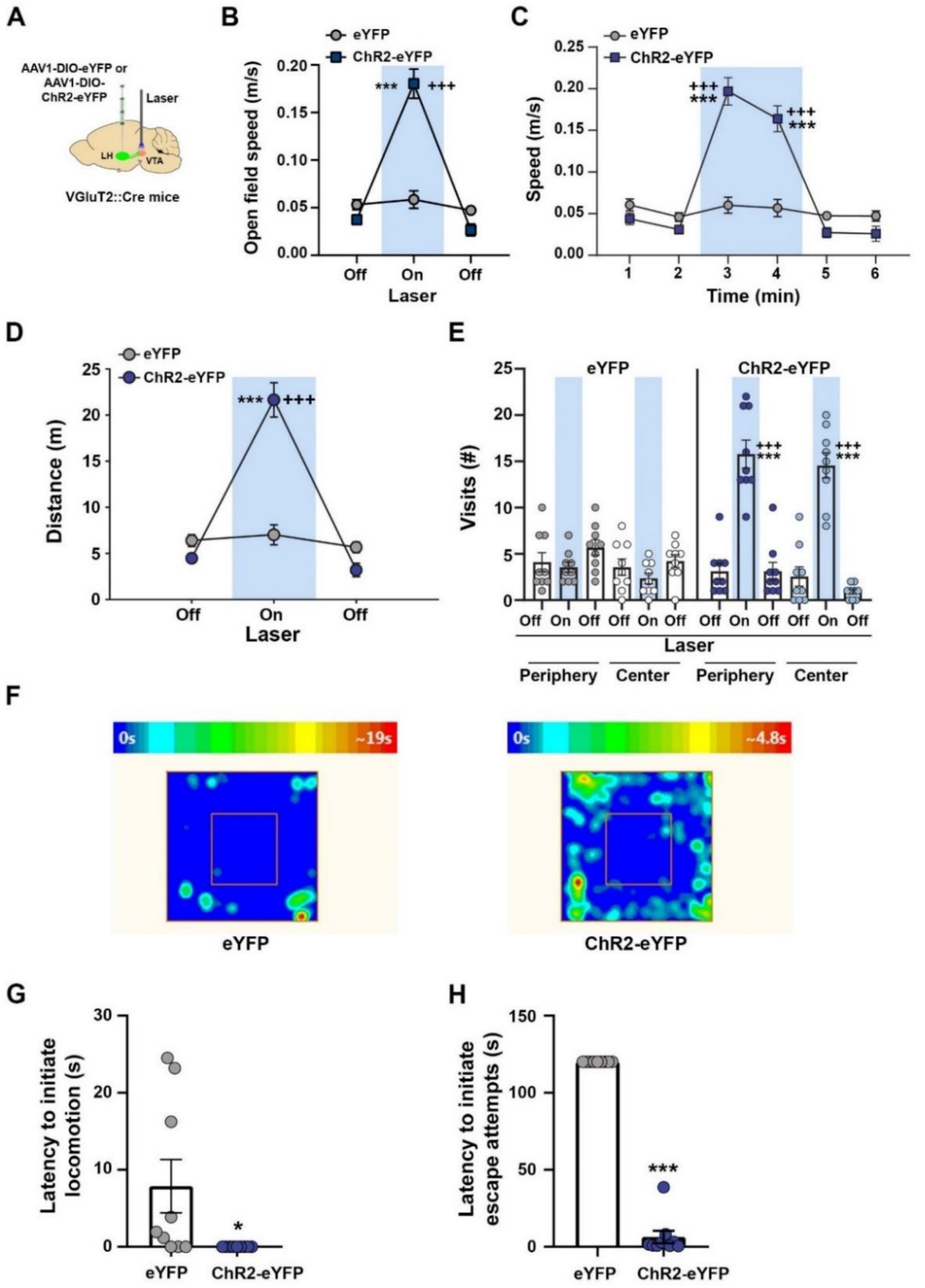

Supplementary Figure 9. VTA photostimulation of LH-VGluT2 fibers increases locomotion and induces escape attempts. A. LH injection of AAV1-DIO-eYFP or AAV1-DIO-ChR2-eYFP and VTA optic fiber. **B.** ChR2-eYFP mice (n=9) moved significantly faster than eYFP control mice (n=9) during VTA photostimulation of LH-VGluT2 fibers (group x experimental phase: $F_{(2,32)}$=48.37, P<0.001, ANOVA with Newman-Keuls post hoc test). **C.** The significant increase in speed showed by ChR2-eYFP mice (n=9) was only observed during VTA photostimulation of LH-VGluT2 fibers (group x time point: $F_{(5,80)}$=37.13, P<0.001, ANOVA with Newman-Keuls post hoc test). Asterisks indicate significant differences between groups (eYFP, n=9), plus signs indicate significant difference with laser off periods. *** P<0.001, +++ P<0.001. **D.** ChR2-eYFP mice (n=9) moved more than eYFP control mice (n=9) during VTA photostimulation of LH-VGluT2 fibers (group x experimental phase: $F_{(2,32)}$=48.23, P<0.001, ANOVA with Newman-Keuls post hoc test). Asterisks indicate significant differences between groups, plus signs indicate significant differences between experimental phases (laser off *vs.* on). *** P<0.001, +++ P<0.001. **E.** The total number of visits to both the periphery and center zones of the open field was increased in ChR2-eYFP mice (n=9) but not in eYFP control mice (n=9) during VTA photostimulation of LH-VGluT2 fibers (group x experimental phase: $F_{(2,32)}$=34.73, P<0.001, ANOVA with Newman-Keuls post hoc test). Asterisks indicate significant differences between groups for the same laser period, plus signs indicate significant differences between experimental phases (laser off *vs.* on) within each group. *** P<0.001, +++ P<0.001. **F.** Heatmaps showing the distance travelled by an eYFP mouse (left) and a ChR2-eYFP mouse (right) during VTA photostimulation of LH-VGluT2 fibers. **G.** ChR2-eYFP mice (n=9) showed shorter latency than eYFP control mice (n=9) to initiate locomotion after VTA photostimulation of LH-VGluT2 fibers started (U=13.50, Z=2.38, * P<0.05, Mann-Whitney U test). **H.** ChR2-eYFP mice (n=9) showed shorter latency than eYFP control mice (n=9) to initiate escape attempts (jumps) VTA photostimulation of LH-VGluT2 fibers started (U=0, Z=3.58, *** P<0.001, Mann-Whitney U test). Light-blue rectangles indicate photostimulation. Data are presented as mean values ± SEM. Source data are provided as a Source Data file.

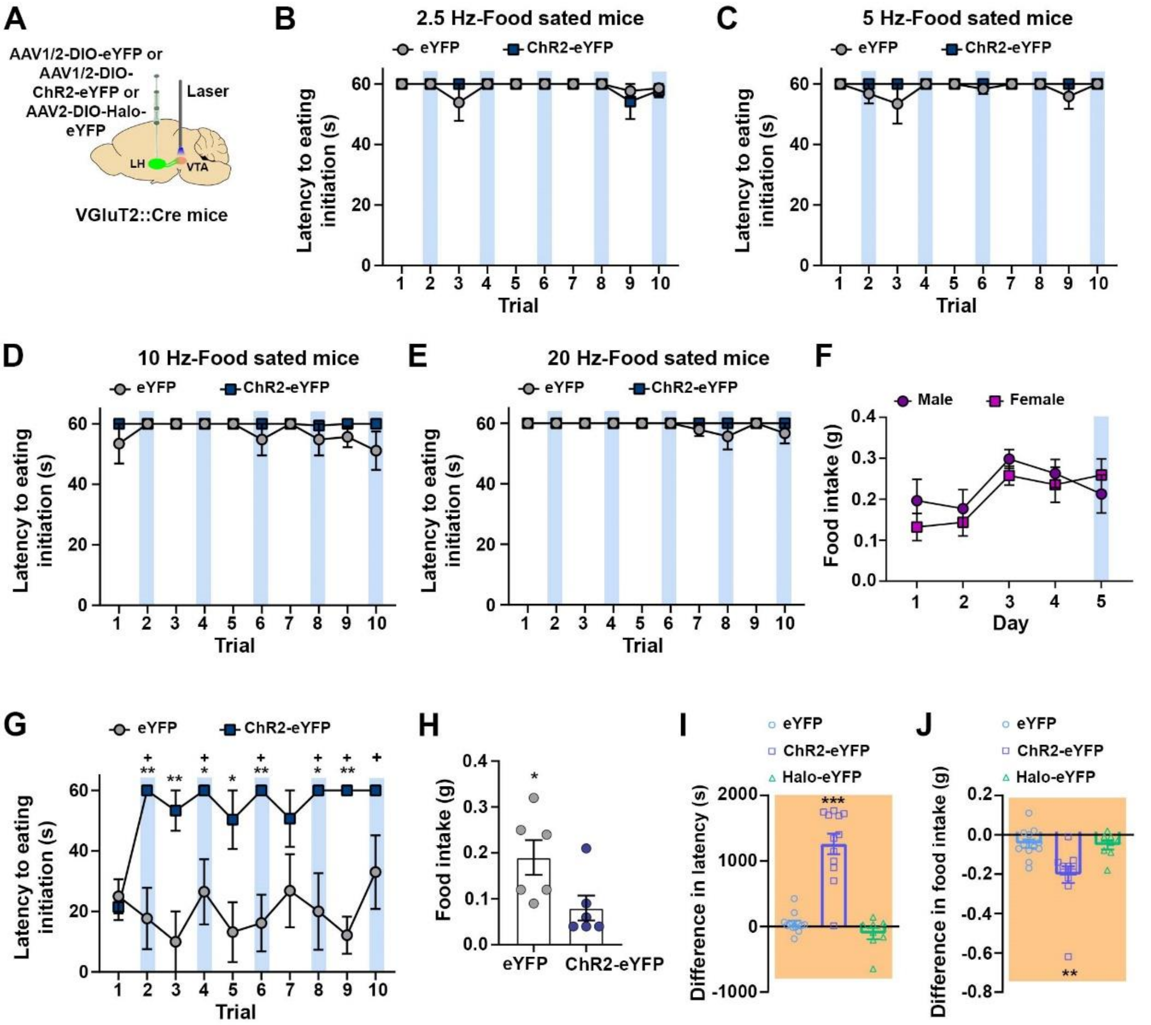

Supplementary Figure 10. VTA photostimulation of LH-VGluT2 fibers disrupts feeding behavior. A. LH injection of AAV1-DIO-eYFP, AAV1-DIO-ChR2-eYFP, AAV2-DIO-eYFP, AAV2-DIO-ChR2-eYFP or AAV2-DIO-Halo-eYFP and VTA optic fiber. **B.** VTA photostimulation of LH-VGluT2 fibers at 2.5 Hz did not elicit feeding in sated eYFP (n=9) or ChR2-eYFP (n=10) mice (eYFP, $X^2_{(9)}$=7.00, P=0.64; ChR2-eYFP, $X^2_{(9)}$=9.00, P=0.44, Friedman ANOVA). **C.** VTA photostimulation of LH-VGluT2 fibers at 5 Hz did not elicit feeding in sated eYFP (n=9) or ChR2-eYFP (n=10) mice (eYFP, $X^2_{(9)}$=6.37, P=0.70; ChR2-eYFP, $X^2_{(9)}$=9.00, P=0.44, Friedman ANOVA). **D.** VTA photostimulation of LH-VGluT2 fibers at 10 Hz did not elicit feeding in sated eYFP (n=9) or ChR2-eYFP (n=10) mice (eYFP, $X^2_{(9)}$=9.30, P=0.41; ChR2-eYFP, $X^2_{(9)}$=9.00, P=0.44, Friedman ANOVA). **E.** VTA photostimulation of LH-VGluT2 fibers at 20 Hz did not elicit feeding in sated eYFP (n=9) or ChR2-eYFP (n=10) mice (eYFP, $X^2_{(9)}$=7.57, P=0.58; ChR2-eYFP, $X^2_{(9)}$=9.00, P=0.44, Friedman ANOVA). **F.** No sex differences were observed in the amount of food eaten by food restricted mice throughout the feeding experiment, either with (day 5) or without (days 1 to 4) VTA photostimulation of LH-VGluT2 fibers (females, n=13; males, n=11; sex x day: $F_{(4,88)}$=0.79, P=0.53, n.s., ANOVA). **G.** Repetitive VTA photostimulation of LH-VGluT2 fibers at 20 Hz increased the latency to initiate eating in food restricted ChR2-eYFP (n=6), but not in food restricted eYFP mice (n=6; group x trial: $F_{(9,90)}$=2.02, P<0.05, ANOVA with Newman-Keuls post hoc test). * P<0.05, ** P<0.01, against eYFP; + P<0.05, against first trial. **H.** Repetitive VTA photostimulation of LH-VGluT2 fibers decreased the amount of food eaten only in food restricted ChR2-eYFP mice (eYFP, n=6; ChR2-eYFP, n=6; $t_{(10)}$=-2.36, P<0.05, two-tailed t test). **I.** ChR2-eYFP mice (n=12) showed a higher latency to eating initiation during VTA laser administration than Halo-eYFP (n=8) or eYFP control mice (n=12) in 30-min experimental sessions (group: $F_{(2,29)}$=46.08, P<0.001, ANOVA with Newman-Keuls post hoc test). *** P<0.001, against eYFP mice. **J.** ChR2-eYFP mice (n=12) ate less during VTA laser administration than Halo-eYFP (n=8) or eYFP control mice (n=12) in 30-min experimental sessions (group: $F_{(2,29)}$=8.16, P<0.01, ANOVA with Newman-Keuls post hoc test). ** P<0.01, against eYFP mice. Light-blue rectangles indicate photostimulation. Orange rectangles indicate either photostimulation or photoinhibition. Data are presented as mean values ± SEM. Source data are provided as a Source Data file.

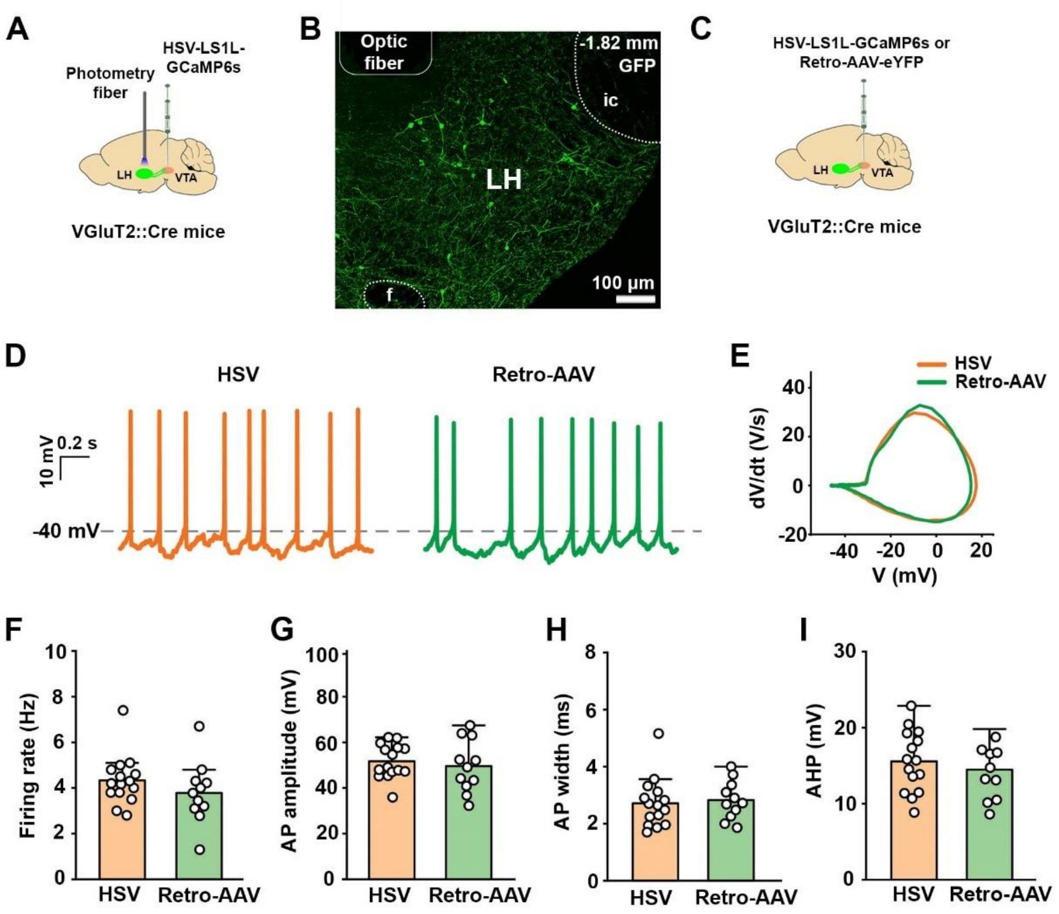

Supplementary Figure 11. HSV-LS1L-GCaMP6s expression does not alter neuronal physiology in LH neurons. A. VTA injection of retrograde vector HSV-LS1L-GCaMP6s and LH photometry fiber. **B.** LH optic fiber placement and detection of GFP in LH-VGluT2 neurons (green). **C.** VTA injection of retrograde vectors HSV-LS1L-GCaMP6s or AAV-eYFP for electrophysiological recordings in LH neurons. **D.** Whole cell current-clamp recordings of spontaneous action potentials (AP) from LH neurons expressing GFP (from HSV-LS1L-GCaMP6s injections, left, orange) or eYFP (from retro-AAV-eYFP injections, right, green). **E.** Overlay of action potentials phase plots from LH neurons shown in **D**. **F.** Firing rate (Hz) of LH neurons expressing GFP (from HSV-LS1L-GCaMP6s injections, n=15, 4.33 ± 0.28 Hz) or eYFP (from retro-AAV-eYFP injections, n=11, 3.78 ± 0.41 Hz); $t_{(24)}$=1.15; p=0.26, two-tailed t test. **G.** AP amplitude (mV) of LH neurons expressing GFP (from HSV-LS1L-GCaMP6s injections, n=15, 51.7 ± 1.94 mV) or eYFP (from retro-AAV-eYFP injections, n=11, 49.57 ± 3.47 mV); $t_{(24)}$=0.57; p=0.57, two-tailed t test. **H**. AP width (ms) of LH neurons expressing GFP (from HSV-LS1L-GCaMP6s injections, n=15, 2.71 ± (2.25×10^{-4}) ms) or eYFP (from retro-AAV-eYFP injections, n=11, 2.83 ± (2.05×10^{-4}) ms); $t_{(24)}$=-0.35; p=0.73, two-tailed t test. **I.** After-hyperpolarization (AHP, mV) of LH neurons expressing GFP (from HSV-LS1L-GCaMP6s injections, n=15, 15.56 ± 1.04 mV) or eYFP (from retro-AAV-eYFP injections, n=11, 14.47 ± 1.11 mV); $t_{(24)}$=0.70; p=0.49, two-tailed t test. Data are presented as mean values ± SEM. Source data are provided as a Source Data file.

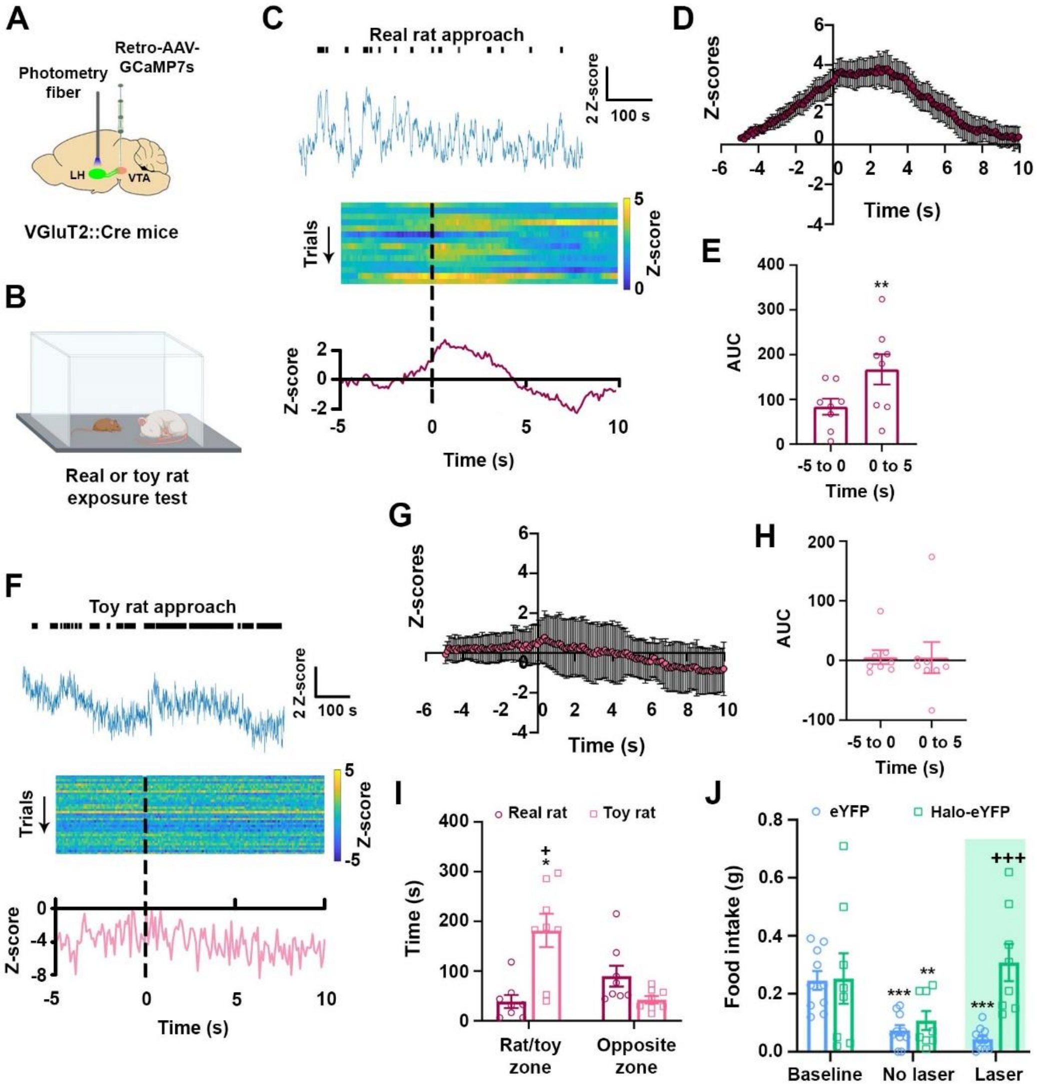

Supplementary Figure 12. LH-VGluT2 neurons innervating the VTA signal the presence of an anesthetized rat but not the presence of a toy rat. A. VTA injection of retrograde vector AAV-GCaMP7s and LH photometry fiber. **B.** Mice were tested in the presence of an anesthetized rat. Created with BioRender.com. **C.** Whole session recording of LH-VGluT2 neurons projecting to the VTA showing approaches to the anesthetized rat (top); heatmap of Ca^{2+} activity over successive rat approach trials (middle); cell population responses to rat approach onset showing increases in Ca^{2+} activity in LH-VGluT2 neurons projecting to the VTA (bottom). **D.** Population Ca^{2+} activity (+SEM) in LH-VGluT2 neurons projecting to VTA during rat approach onset (n=8). **E.** AUC for Ca^{2+} activity in LH-VGluT2 neurons projecting to VTA before (−5 to 0 s) and after (0 to 5 s) onset of rat approach (n=8; $t_{(7)}$=-4.60, * P<0.01, two-tailed t test). **F.** Whole session recording of LH-VGluT2 neurons projecting to the VTA showing approaches to a toy rat (top); heatmap of Ca^{2+} activity over successive toy rat approach trials (middle); cell population responses to toy rat approach onset showing increases in Ca^{2+} activity in LH-VGluT2 neurons projecting to the VTA (bottom). **G.** Population Ca^{2+} activity (+SEM) in LH-VGluT2 neurons projecting to VTA during toy rat approach onset (n=8). **H.** AUC for Ca^{2+} activity in LH-VGluT2 neurons projecting to VTA before (−5 to 0 s) and after (0 to 5 s) onset of toy rat approach (n=8; $t_{(7)}$=-4.60, * P<0.01, two-tailed t test). I. Mice explored more the toy rat (expressed as more time spent in the toy rat zone and less time spent in the zone opposite to the toy rat) than the anesthetized rat (n=8, stimulus x zone: $F_{(1,17)}$=16.28, P<0.01, ANOVA with Newman-Keuls post hoc test). * P<0.05, against real rat for the same zone; + P<0.05, against opposite zone for the same stimulus. **J.** Food intake was significantly reduced in Halo-eYFP (n=8) and eYFP control mice (n=10) when presented with an anesthetized rat in 30-min experimental sessions. Photoinhibition restored feeding to baseline levels in Halo-eYFP mice (group x experimental phase: $F_{(2,32)}$=12.91, P<0.001, ANOVA with Newman-Keuls post hoc test). ** P<0.01, *** P<0.001, against baseline, +++ P<0.00, against eYFP. Green rectangle indicates photoinhibition. Data are presented as mean values ± SEM. Source data are provided as a Source Data file.

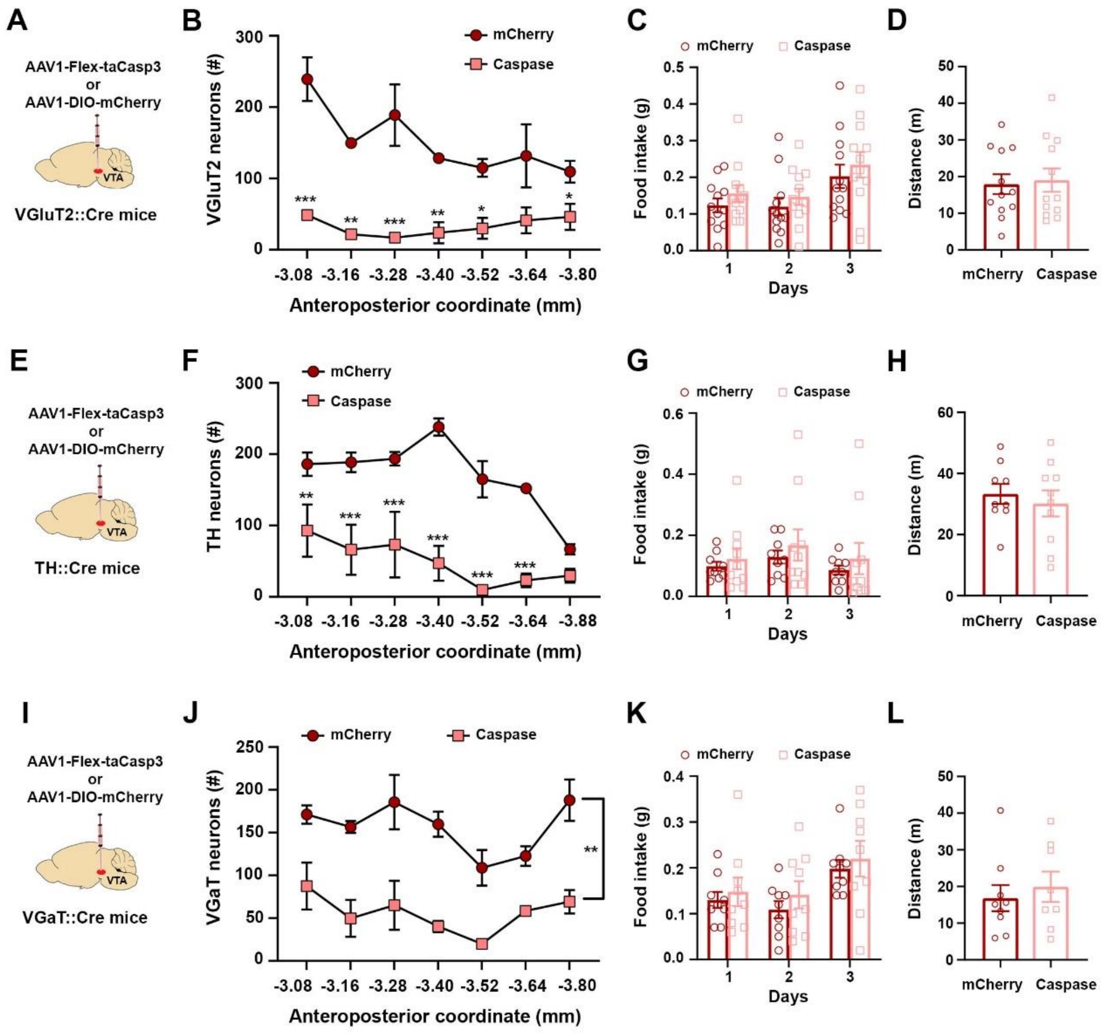

Supplementary Figure 13. Anteroposterior distribution of VTA-VGluT2, VTA-TH and VTA-VGaT neurons, basal food intake and locomotion in control and genetically ablated mice. A. VTA injection of AAV1-Flex-taCasp3 or AAV1-DIO-mCherry in VGluT2::Cre mice. **B.** VGluT2 neurons are present in mCherry control mice but are infrequent in caspase mice at different anteroposterior levels of the VTA (group x coordinate: $F_{(6,24)}$=2.68, P<0.05, ANOVA with Newman-Keuls post hoc test). * P<0.05, ** P<0.01, *** P<0.001, against control mice. **C.** mCherry (n=12) and VTA-VGluT2 caspase-ablated (n=12) mice ate similar amounts of food during the three days prior to the rat exposure test (group x day: $F_{(2,44)}$=0.01, P=0.99, n.s., ANOVA). **D.** mCherry (n=12) and VTA-VGluT2 caspase-ablated (n=12) mice travelled similar distances during the rat exposure test ($t_{(22)}$=-0.27; P=0.79, n.s., two-tailed t test). **E.** VTA injection of AAV1-Flex-taCasp3 or AAV1-DIO-mCherry in TH::Cre mice. **F.** TH neurons are present in mCherry control mice but are infrequent in caspase mice at different anteroposterior levels of the VTA (group x coordinate: $F_{(6,24)}$=4.72, P<0.05, ANOVA with Newman-Keuls post hoc test). ** P<0.01, *** P<0.001, against control mice. **G.** mCherry (n=9) and VTA-TH caspase-ablated (n=10) mice ate similar amounts of food during the three days prior to the rat exposure test (group x day: $F_{(2,34)}$=0.15, P=0.86, n.s., ANOVA). **H.** mCherry (n=9) and VTA-TH caspase-ablated (n=10) mice travelled similar distances during the rat exposure test ($t_{(17)}$=0.57; P=0.58, n.s., two-tailed t test). **I.** VTA injection of AAV1-Flex-taCasp3 or AAV1-DIO-mCherry in VGaT::Cre mice. **J.** VGaT neurons are present in mCherry control mice but are infrequent in caspase mice at different anteroposterior levels of the VTA (main effect group: $F_{(1,4)}$=59.29, P<0.01, ANOVA with Newman-Keuls post hoc test). ** P<0.01, against control mice. **K.** mCherry (n=9) and VTA-TH caspase-ablated (n=8) mice ate similar amounts of food during the three days prior to the rat exposure test (group x day: $F_{(2,30)}$=0.17, P=0.85, n.s., ANOVA). **H.** mCherry (n=9) and VTA-TH caspase-ablated (n=8) mice travelled similar distances during the rat exposure test ($t_{(15)}$=0.57; P=0.58, n.s., two-tailed t test). Data are presented as mean values ± SEM. Source data are provided as a Source Data file.

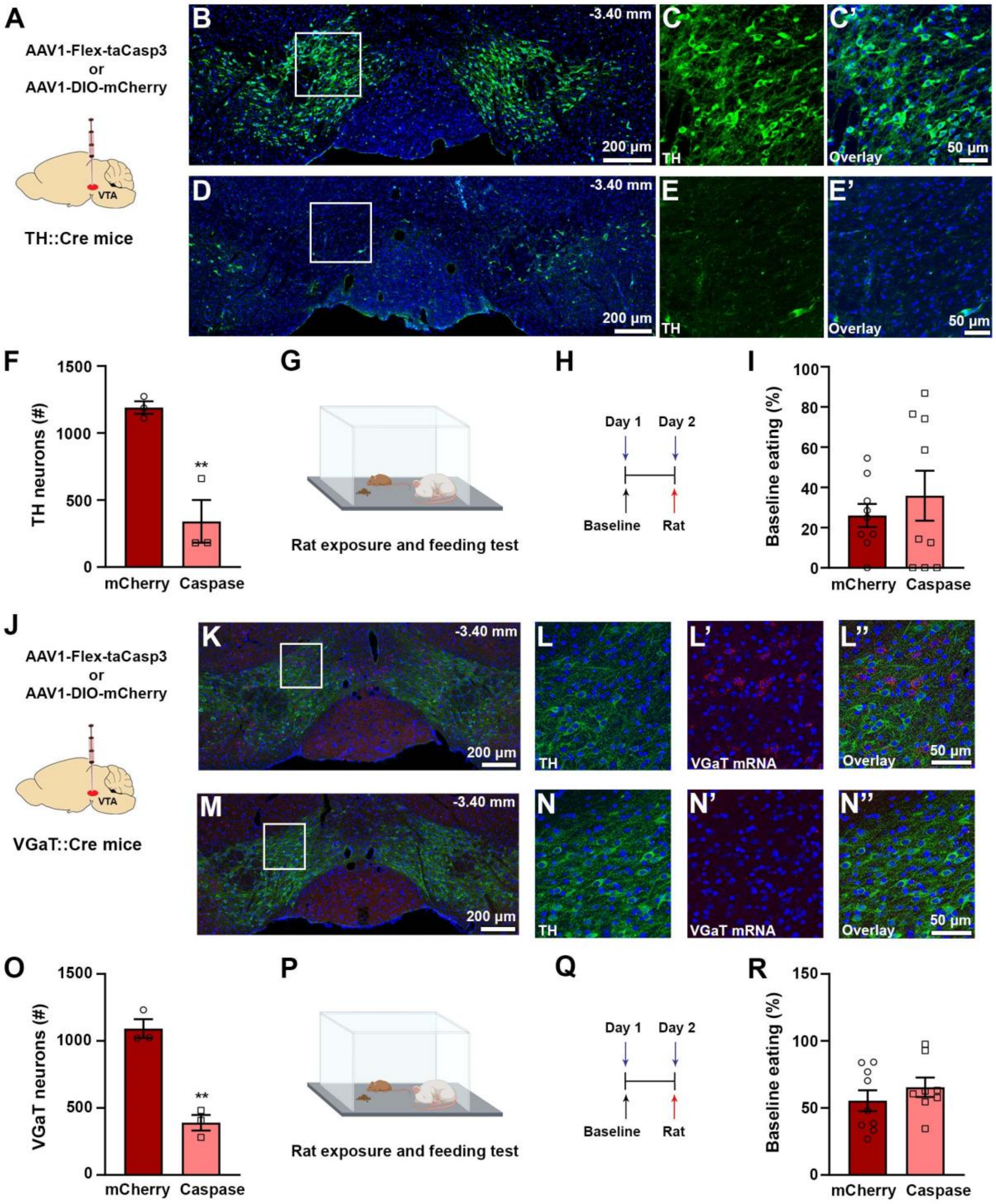

Supplementary Figure 14. VTA-TH or VGaT neurons do not play a role in feeding disruption resulting from the presence of a predator. A. VTA injection of AAV1-Flex-taCasp3 or AAV1-DIO-mCherry. **B–C'.** Low (**B**) and high (**C-C'**) magnification of VTA from a control mouse (injected with AAV1-DIO-mCherry) showing neurons expressing TH-immunoreactive neurons (TH-IRs; green). **D-E'.** Low (**D**) and high (**E–E'**) magnification of VTA from a mouse injected with AAV1-Flex-taCasp3 showing lack of TH-IRs. **F.** VTA-TH neurons are present in control mice (1189.94 ± 47.03; 3 mice) but are infrequent in caspase mice (341.17 ± 159.58; 3 mice; 7 sections per mouse; $t_{(4)}$=5.10, two-tailed t test). ** P<0.01, against control mice. **G-H.** Food restricted caspase and control mice exposure to both anesthetized rat and food (**G**, created with BioRender.com) and test timeline (**H**). **I.** Food intake was significantly reduced in both control (n=9) and caspase mice (n=9) when presented with an anesthetized rat ($t_{(16)}$=-0.72, p=0.48, n.s., two-tailed t test). **J.** VTA injection of AAV1-Flex-taCasp3 or AAV1-DIO-mCherry. **K–L''.** Low (**K**) and high (**L-L''**) magnification of VTA from a control mouse (injected with AAV1-DIO-mCherry) showing neurons expressing VGaT mRNA (red) intermixed with TH-IRs (green). **M-N''.** Low (**M**) and high (**N–N''**) magnification of VTA from a mouse injected with AAV1-Flex-taCasp3 showing TH-IRs and lack of VGaT mRNA. **O.** VTA-VGaT neurons are present in control mice (1092.22 ± 69.90; 3 mice) but are infrequent in caspase mice (390.06 ± 58.57; 3 mice; 7 sections per mouse; $t_{(4)}$ = 7.70, two-tailed t test). ** P<0.01, against control mice. **P-Q.** Food restricted caspase and control mice exposure to both anesthetized rat and food (**P**, created with BioRender.com) and test timeline (**Q**). **R.** Food intake was significantly reduced in both control (n=9) and caspase mice (n=8) when presented with an anesthetized rat ($t_{(15)}$=-0.93, p=0.36, n.s., two-tailed t test). Data are presented as mean values ± SEM. Source data are provided as a Source Data file.
