# Supplementary material for: Lateral hypothalamic glutamatergic inputs to VTA glutamatergic neurons mediate prioritization of innate defensive behavior over feeding
Source: Nat Commun. 2024 Jan 9;15:403. doi: 10.1038/s41467-023-44633-w (PMC10776608; doi:10.1038/s41467-023-44633-w)
Supplement: Supplementary file 3 — Inventory of Additional Supplementary Information [file 41467_2023_44633_MOESM3_ESM.pdf]

Inventory of supporting information

-Supplementary information file (2 tables and 14 figures)

-Editorial policy checklist

-Third-party rights table

-Publication license agreements for BioRender.com

-Author’s checklist completed

-Source data file

-Reporting summary
