# Supplementary material for: Lateral hypothalamic glutamatergic inputs to VTA glutamatergic neurons mediate prioritization of innate defensive behavior over feeding
Source: Nat Commun. 2024 Jan 9;15:403. doi: 10.1038/s41467-023-44633-w (PMC10776608; doi:10.1038/s41467-023-44633-w)
Supplement: Supplementary file 4 — Reporting Summary [file 41467_2023_44633_MOESM4_ESM.pdf]

nature portfolio

Corresponding author(s): Dr. Marisela Morales

Last updated by author(s): Dec 11, 2023

Reporting Summary

Nature Portfolio wishes to improve the reproducibility of the work that we publish. This form provides structure for consistency and transparency in reporting. For further information on Nature Portfolio policies, see our Editorial Policies and the Editorial Policy Checklist.

Statistics

For all statistical analyses, confirm that the following items are present in the figure legend, table legend, main text, or Methods section.

n/a	Confirmed	
☐	☒	The exact sample size (*n*) for each experimental group/condition, given as a discrete number and unit of measurement
☒	☐	A statement on whether measurements were taken from distinct samples or whether the same sample was measured repeatedly
☐	☒	The statistical test(s) used AND whether they are one- or two-sided<br>*Only common tests should be described solely by name; describe more complex techniques in the Methods section.*
☒	☐	A description of all covariates tested
☒	☐	A description of any assumptions or corrections, such as tests of normality and adjustment for multiple comparisons
☐	☒	A full description of the statistical parameters including central tendency (e.g. means) or other basic estimates (e.g. regression coefficient) AND variation (e.g. standard deviation) or associated estimates of uncertainty (e.g. confidence intervals)
☐	☒	For null hypothesis testing, the test statistic (e.g. *F*, *t*, *r*) with confidence intervals, effect sizes, degrees of freedom and *P* value noted<br>*Give P values as exact values whenever suitable.*
☒	☐	For Bayesian analysis, information on the choice of priors and Markov chain Monte Carlo settings
☒	☐	For hierarchical and complex designs, identification of the appropriate level for tests and full reporting of outcomes
☒	☐	Estimates of effect sizes (e.g. Cohen's *d*, Pearson's *r*), indicating how they were calculated

Our web collection on statistics for biologists contains articles on many of the points above.

Software and code

Policy information about availability of computer code

Data collection	Behavioral data was acquired with video tracking system Anymaze (version 7.0, Stoelting). Photometry recordings were made with Synapse software (version 96, TDT). Microscope images were acquired with Zen software (version 3.1, Zeiss).
Data analysis	Statistical analyses were performed using Statistica software (Cloud Software Group Inc.). Photometry results were analyzed with pMAT scripts (Open source, the Barker lab, https://www.thebarkerlab.com/resources). Electrophysiological recordings were analyzed with pClamp 11 software suite (version 11.2, Molecular Devices).

For manuscripts utilizing custom algorithms or software that are central to the research but not yet described in published literature, software must be made available to editors and reviewers. We strongly encourage code deposition in a community repository (e.g. GitHub). See the Nature Portfolio guidelines for submitting code & software for further information.

Data

Policy information about availability of data

All manuscripts must include a data availability statement. This statement should provide the following information, where applicable:

- Accession codes, unique identifiers, or web links for publicly available datasets
- A description of any restrictions on data availability
- For clinical datasets or third party data, please ensure that the statement adheres to our policy

The source data generated in this study have been deposited in the Zenodo repository under DOI 10.5281/zenodo.10357212 (https://zenodo.org/doi/10.5281/

zenodo.10357211)

Research involving human participants, their data, or biological material

Policy information about studies with human participants or human data. See also policy information about sex, gender (identity/presentation), and sexual orientation and race, ethnicity and racism.

Reporting on sex and gender	Not applicable
Reporting on race, ethnicity, or other socially relevant groupings	Not applicable
Population characteristics	Not applicable
Recruitment	Not applicable
Ethics oversight	Not applicable

Note that full information on the approval of the study protocol must also be provided in the manuscript.

Field-specific reporting

Please select the one below that is the best fit for your research. If you are not sure, read the appropriate sections before making your selection.

☒ Life sciences ☐ Behavioural & social sciences ☐ Ecological, evolutionary & environmental sciences

For a reference copy of the document with all sections, see nature.com/documents/nr-reporting-summary-flat.pdf

Life sciences study design

All studies must disclose on these points even when the disclosure is negative.

Sample size	No statistical methods were used to predetermine sample size, but sample sizes are consistent with those reported in previous publications in the field and from our laboratory (Barker et al., 2023; Barbano et al., 2020; Root et al., 2020).
Data exclusions	In optogenetic experiments, mice with fiber tip placement outside of the target structure were excluded from the analysis.
Replication	All the experiments were successfully repeated three times to ensure reproducibility of the results and all attempts at replication were successful.
Randomization	Surgical and behavioral manipulations performed on each animal were randomly determined. In the case of behavioral experiments, the viral vector injected was determined randomly and counterbalanced across animals. Mice from each litter were randomly assigned into treatment groups.
Blinding	All experiments were conducted in a blind manner such that assays were conducted and analyzed without knowledge of the experimental group of the animal under study.

Reporting for specific materials, systems and methods

We require information from authors about some types of materials, experimental systems and methods used in many studies. Here, indicate whether each material, system or method listed is relevant to your study. If you are not sure if a list item applies to your research, read the appropriate section before selecting a response.

Materials & experimental systems

n/a	Involved in the study	
☐	☒	Antibodies
☒	☐	Eukaryotic cell lines
☒	☐	Palaeontology and archaeology
☐	☒	Animals and other organisms
☒	☐	Clinical data
☒	☐	Dual use research of concern
☒	☐	Plants

Methods

n/a	Involved in the study	
☒	☐	ChIP-seq
☒	☐	Flow cytometry
☒	☐	MRI-based neuroimaging

Antibodies

Antibodies used	Primary antibodies: rabbit anti-GFP (Frontier Institute, GFP-Rb-Af2020, 1:2000), mouse anti-GFP (Takara, 632381, 1:500), mouse anti-TH (Millipore Sigma, MAB318, 1:1000), mouse anti-mCherry (1:1,000, Takara, 632543), rabbit anti–cFos (1:300, Santa Cruz Biotechnology, SC-52).<br>Secondary antibodies: biotinylated goat anti-rabbit (Vector Laboratories, BA-1000, 1:200), Alexa-Fluor-594 donkey anti-mouse (Jackson ImmunoResearch, 715-585-151, 1:100), Alexa-Fluor-488 donkey anti-rabbit (Jackson ImmunoResearch, 711-545-152, 1:100), Alexa-Fluor-488 donkey anti-mouse (Jackson ImmunoResearch, 715-545-150, 1:100), biotinylated goat anti-mouse (Vector Laboratories, BA-9200, 1:100).
Validation	All the antibodies used are commercially available and already validated (see manufacturer's websites). Rabbit anti-GFP: validated in a GFP transgenic mouse line, as per manufacturer's 20221027 data sheet (no staining observed in wild-type mice, reference: Takasaki et al., 2010, Eur. J. Neurosci.). Mouse anti-GFP: validated by Western blot analysis using lysate made from a HEK 293 cell line stably expressing AcGFP1. A band of approximately 30 kDa corresponding to AcGFP1 was observed in the lane loaded with the AcGFP1 cell lysate. A band of this size was not detected in the lysate of untransfected HEK 293 cells. Mouse anti-TH: validated by Western blot using lysate from PC12 cells or mouse brain lysates. Mouse anti-mCherry: validated by Western blot analysis using lysate made from HEK 293 cells transiently transfected with a CMV-driven expression vector encoding mCherry. A band of approximately 30-35 kDa in the lane loaded with the lysate of the transiently transfected HEK 293 cells expressing mCherry. No band in this molecular weight range was detected in the lysate of the untransfected HEK 293 cells. Rabbit anti-cFos: validated by Western blot analysis of cFos expression in HeLa, MIAPaCa-2, NIH/3T3, A-431, whole cell lysates and NIH/3T3 nuclear extracts by the presence of a band of 62 kDa.

Animals and other research organisms

Policy information about studies involving animals; ARRIVE guidelines recommended for reporting animal research, and Sex and Gender in Research

Laboratory animals	Male and female VGluT2::IRES::Cre mice (Slc17a6tm2(cre)Lowl/J, in C57BL/6J background from The Jackson Laboratories, Bar Harbor, ME), VGaT::Cre mice (Slc32a1tm2(cre)Lowl/J, in C57BL/6J background from The Jackson Laboratories) or TH::Cre mice (Thtm1(cre)Te/J, in C57BL/6J background from The Jackson Laboratories) were bred in the NIDA/IRP animal facility and were used in behavioral and anatomical studies. Groups of 2-5 mice (weighing 20-30 g and at least 8 weeks old at the start of experiments) were housed in an animal vivarium maintained on a direct 12-h light-dark cycle (lights on at 7:00 am) at a constant temperature of 23°C and xx humidity. Long Evans rats (at least 8 weeks old, obtained from the NIDA/IRP breeding facility) were used for the rat exposure test.
Wild animals	The study did not involve wild animals.
Reporting on sex	Male and female mice were used in our study. Sex as a variable was analyzed in one of the experiments but, due to the lack of effect, was not further analyzed in the other experiments.
Field-collected samples	The study did not involve samples collected from the field.
Ethics oversight	Animal Care and Use Committee of the National Institute on Drug Abuse

Note that full information on the approval of the study protocol must also be provided in the manuscript.

Plants

Seed stocks	Not applicable
Novel plant genotypes	Not applicable
Authentication	Not applicable
